# Comprehensive analysis of structure–property–shielding relationships in Pr_2_O_3_-modified tellurite glass systems

**DOI:** 10.1038/s41598-025-29373-9

**Published:** 2025-12-09

**Authors:** M. S. Gaafar, S. Y. Marzouk, H. M. Elsaghier, Eman A. Mwafy

**Affiliations:** 1 National Institute of Standards, Tersa Str, P.O. Box 136, El-Haram, El-Giza, 12211 Egypt; 2 Basic and Applied Science Department, College of Engineering and Technology, Arab Academy for Science, Technology, and Maritime Transport, Al-Horria, Heliopolis, Cairo Egypt; 3 Engineering Physics and Mathematics Department, Faculty of Engineering – Mataria, Helwan University, Cairo, Egypt; 4 Physical Chemistry Department, Institute of Advanced Materials Technology and Mineral Resources, National Research Centre, Cairo, 12622 Egypt

**Keywords:** Tellurite glasses, Radial distribution function, FTIR, Mechanical and thermal properties, Shielding parameters, Chemistry, Materials science, Physics

## Abstract

This work investigated the structural, thermal, mechanical, and shielding characteristics of a tellurite-based glass series with the composition 60TeO_2_–12.5Nb_2_O_5_–12.5ZnO–(15–*x*)LiF–*x*Pr_2_O_3_, where *x* ranged from 0.5 to 5.0 mol%. The novelty of this study lay in a comprehensive analysis that combined multiple techniques. The research involved using the radial distribution function (RDF) with Gaussian fitting to determine structural parameters and the fraction of trigonal bipyramidal (TeO_4_), N_4_(X-ray) representation for each glass sample. The study also utilized deconvoluted FTIR spectra to further characterize the glass structure. Results showed the transformation of trigonal pyramidal (TeO_3_) tp units to trigonal bipyramidal (TeO_4_) tbp units and consequently enhanced the rigidity of the glass structure with addition of Pr_2_O_3_. Additionally, it established a logarithmic correlation across the entire glass series, linking the molar volume (V_m_) with the experimental bulk modulus (K_exp_​), $\:{K}_{exp}={V}_{m}^{-\alpha\:}$, and explored the correlations between these properties and the power (α). The ring deformation model was applied to calculate the average atomic ring diameter. Increased Pr_2_​O_3_​ content enhanced the glass’s thermal stability. This was evidenced by a significant rise in its glass transition temperature and onset crystallization temperature, indicating a more rigid and stable structure. In addition, various shielding parameters were determined. The results of this investigation highlight the excellent gamma photon shielding capabilities of these glasses.

## Introduction

Tellurite (TeO_2_​) glass offers distinct advantages over traditional silicate (SiO_2_​​) glass, primarily due to its high polarizability and refractive index^1^. These characteristics make it possible to create “multifunction” or “smart” glasses through doping^2,3^. Tellurite glasses are now being recognized as versatile, high-performance materials for ionizing radiation shielding. Their unique combination of high density, excellent optical performance, and adaptable composition makes them ideal for diverse applications^4–8^. Furthermore, they are a more environmentally friendly and lighter alternative to conventional shielding options like lead or concrete and can be precisely tailored to specific application requirements^9–11^.

Incorporating modifying oxides like zinc oxide (ZnO), niobium oxide (Nb_2_​O_5_​), and lithium fluoride (LiF) is crucial for enhancing glass’s physical and chemical stability. In addition, the introduction of rare-earth oxides, specifically praseodymium oxide (Pr_2_​O_3_​), leads to substantial alterations in the glass’s structural, optical, and electrical properties. Praseodymium-doped glasses are particularly valuable for photonic devices like fiber lasers, optical amplifiers, and illuminating materials because praseodymium ​ ion creates active luminous centers that enhance emission properties. The presence of Pr_2_​O_3_​ also modifies the glass’s bonding environment, boosts its overall durability, and changes its glass transition temperature^12^.

A comprehensive study was conducted to explore the potential of a new tellurite glass, 70TeO_2_–15ZnO–6.5La_2_O_3_–8.5WO_3_ (TZLW), for use in acousto-optic applications, particularly in the visible and mid-infrared spectrum^13^. The investigation revealed that this glass has exceptional optical and thermal properties, including high optical homogeneity (Δn = 5.941 × 10^− 4^), low hydroxyl absorption, high glass transition temperature of 389 °C, excellent thermal stability, and a high Vickers hardness (390.3 kg/mm^2^). Its structural composition, determined through Raman analysis, consists of various tellurium and tungsten oxide units [TeO_4_], [TeO_3_], [WO_4_], and [WO_6_] which contribute to its properties. Notably, the TZLW glass boasts a remarkable acousto-optic figure of merit of 37.2 × 10^− 15^ s^3^/kg at 1550 nm, surpassing all other reported tellurite glasses and commercial quartz by a significant margin. Additionally, its laser-induced damage threshold (LIDT) is 0.572 J/cm^2^ at 800 nm, making it approximately four times more resistant to laser damage than commercial As_2_S_3_ glass.

This inquiry investigates the correlation between radiation shielding and structural properties of 60TeO_2_–12.5Nb_2_O_5_–12.5ZnO–(15–*x*)LiF–*x*Pr_2_O_3_ glasses, with *x* ranging from 0.5 to 5.0 mol %. This is to produce echo friendly, lead-free glasses with high thermal and mechanical stability for gamma rays shielding. Using a multi-faceted approach that includes Fourier-transform infrared spectroscopy (FTIR), radial distribution function (RDF), ultrasonic measurements, and differential scanning calorimetry (DSC), the study offers a detailed view of the glasses’ atomic and molecular configurations. Furthermore, statistical analyses, such as ANOVA, along with calculations from the bond compression and Makishima-Mackenzie models, are used to determine key structural parameters like the structural sensitivity factor, mean cross-link density, and network bond count, providing a comprehensive evaluation of the glasses’ characteristics.

## Experimental procedures

**Fig. 1:**
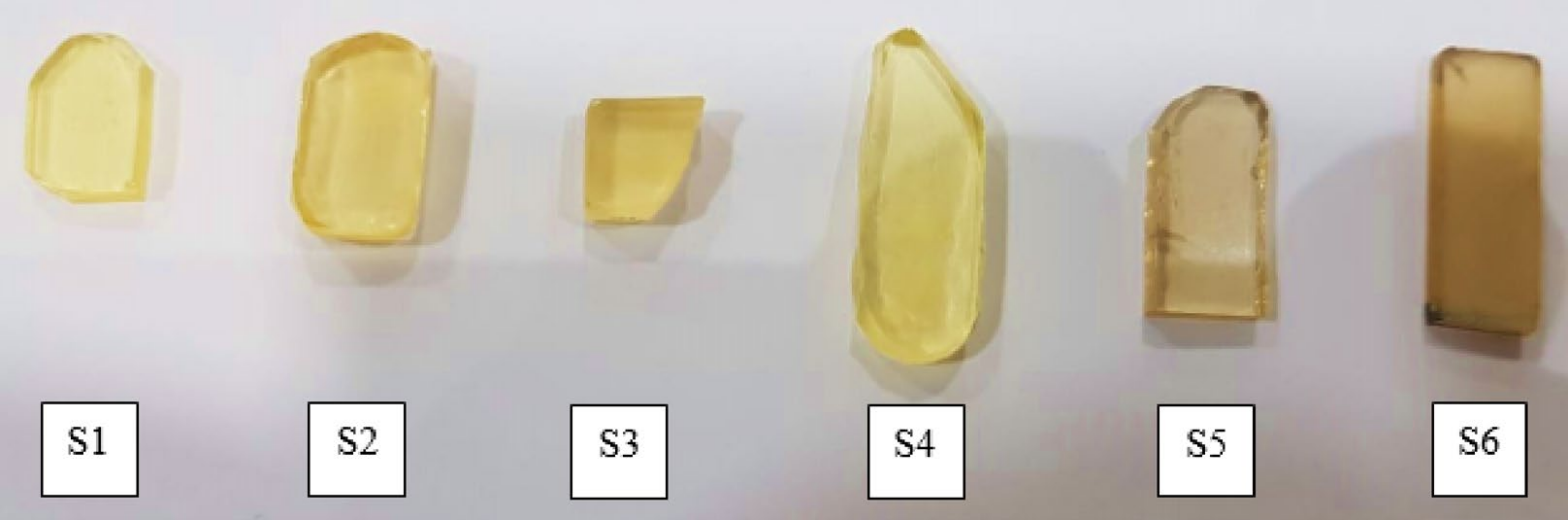
Digital photograph of the synthesized glass samples.

 The rapid quenching method was used to prepare the 60TeO_2_–12.5Nb_2_O_5_–12.5ZnO–(15–*x*)LiF–*x*Pr_2_O_3_ glasses, with *x* values ranging from 0.5 to 5.0 mol %. The batches for each glass composition are presented in Table 1. To begin, the Sigma Aldrich constituent chemicals, all with a purity greater than 99.9%, were weighed in powder form and thoroughly mixed in an agate mortar to ensure homogeneity. This mixture was then preheated at 373 K for an hour to eliminate any residual water. The preheated mixture then melted in an electric furnace at 1173 K for one hour, with intermediate stirring to obtain a homogeneous, bubble-free liquid. The molten glass was then swiftly poured into a preheated mild steel mold (pre heated at 623 K) for melt quenching to obtain the short-range order of the glass structure and left to anneal for another hour at the same temperature to remove the internal stresses and keep remaining the glass structure. This process resulted in solid, bulk glass samples, each measuring approximately 1 × 1 × 1 cm^3^. Figure 1 shows a digital photograph of the samples that were prepared and studied; they were originally presented in an earlier publication^12^.

**Table 1:** Glass compositions in mol %, oxygen to tellurium ions ratio (O/Te), dissociation energy (G_t_), density (ρ), molar volume (V_m_), and their statistical ANOVA results.

Sample code	TeO_2_	Nb_2_O_5_	ZnO	LiF	Pr_2_O_3_	O/Te	G_t_ kJ/mol	ρ kg/m^3^	Z_cal_	Vm m^3^/ (kg.mol)	Z_cal_
**S1**	60	12.5	12.5	14.5	0.5	3.275	57.71	4800	−2.93	0.0301	−2.95
**S2**	60	12.5	12.5	14	1	3.300	57.75	4820	−2.22	0.0303	−2.23
**S3**	60	12.5	12.5	13	2	3.350	57.84	4858	−0.82	0.0307	−0.80
**S4**	60	12.5	12.5	12	3	3.400	57.94	4896	0.59	0.0311	0.61
**S5**	60	12.5	12.5	11	4	3.450	58.03	4935	1.99	0.0315	2.00
**S6**	60	12.5	12.5	10	5	3.500	57.71	4973	3.39	0.0318	3.36
**Mean**								4880		0.0309	
**STDEV**								67		0.0007	

To accurately measure ultrasonic velocity, each glass sample was carefully prepared. The two opposite faces of each sample were ground to be parallel using SiC abrasives, then polished with fine alumina abrasive and machine oil, resulting in a thickness variation of no more than ±20 μm. The ultrasonic velocity was determined using the pulse-echo technique. A transducer sent an ultrasonic wave into the glass sample, and a flaw detector (USN60) captured the reflected wave, or echo. The velocity was then computed by dividing twice the sample’s thickness by the time it took for the wave to travel through it and back. All velocity measurements in this study were carried out at 4 MHz frequency, and at room temperature of 298 K. The estimated error in velocity measurements was ± 6 m/s for longitudinal velocity and ± 3 m/s for shear velocity.

The amorphous structure of all samples was verified by X-ray diffraction (XRD) measurements. The XRD data for all glass compositions were collected using Rigaku Mini-Flex 600 diffractometer with Cu Kα-radiation with step-scanning mode from 4º to 100º (2θ) with a 0.02º step size. The measured intensities underwent standard corrections^14,15^ for Compton scattering, polarization, and absorption. Normalization was implemented using both the Norman and Krogh-Moe methods. The corrected intensities were then used to compute radial distribution function and reduced structural factor, i(Q), where the scattering vector S(Q) is defined as 4πsinθ/λ with λ = 1.542 Å. All calculations followed established procedures from the literature^16–21^. The RDF, g(r) = 4πr^2^ρ_o_G(r), where ρ_o_ is the atomic number density, was obtained by normalizing the pair-correlation function G(r), which was obtained via Fourier transformation of S(Q). Te–O, Te–F, and Pr–O structural links in the glass matrix could be quantitatively assessed according to the average interatomic distances (r) and coordination numbers (N) by the locations of the first and second coordination shells in the RDF curves.

Thermal analysis, including the measurement of the glass transition temperature (T_g_), melting temperature (T_m_), and onset crystallization temperature (T_c_), was carried out on all samples via differential scanning calorimetry (DSC). These thermal studies utilized a Shimadzu DSC-50 (Japan) and involved heating 5–10 mg of powdered samples under N_2_ atmosphere from room temperature to 964 K at a rate of 10 K/min. The T_g_ measurement is reported with an accuracy of ± 2 K.

The density (*ρ*) of all glass samples was determined using the Archimedes principle, with distilled water serving as the immersion fluid. A digital weighing sensitive balance Mettler H 72 (Switzerland) with sensitivity of 0.0001 g/cm^3^ was used to weigh the samples. The experiment was repeated three times, and the error in density measurement in all glass samples is ± 5 kg/m^3^.

The infrared spectra of the glass samples were measured using a JASCO FT/IR–430 spectrometer. The measurements were taken across wavenumber range of 350 to 1100 cm^− 1^. To better understand the structural changes in the glasses, the recorded spectra were deconvoluted.

The elastic properties, including the longitudinal (L), bulk (K), shear (G), and Young’s (E) moduli, along with the Debye temperature (θ_D_​) and Poisson’s ratio (σ), were determined for these glasses doped with various concentrations of Pr_2_​O_3_​, as detailed in references^2,22–29^. The uncertainty values in the elastic moduli were ± 0.12, ± 0.25, ± 0.26, and ± 0.11 GPa for (*K*), (*E*), (L), and (*G*), respectively.

## Results and discussion

### Fourier transform infrared (FTIR) spectra

**Fig. 2:**
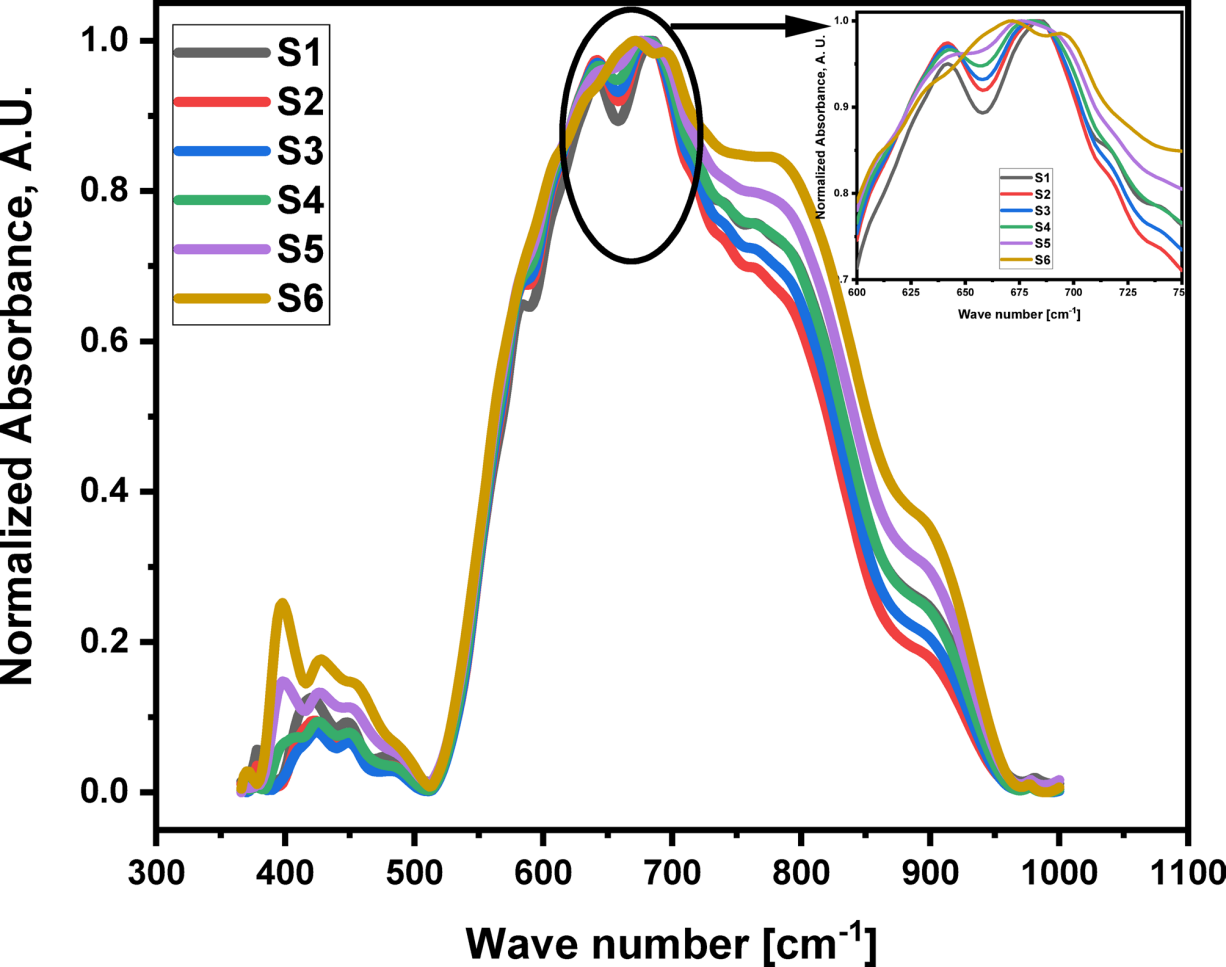
FTIR spectra of 60TeO_2_–12.5Nb_2_O_5_–12.5ZnO–(15–x)LiF–xPr_2_O_3 _glass system in the wave number range from 350 to 1000 cm^-1^.

**Fig. 3:**
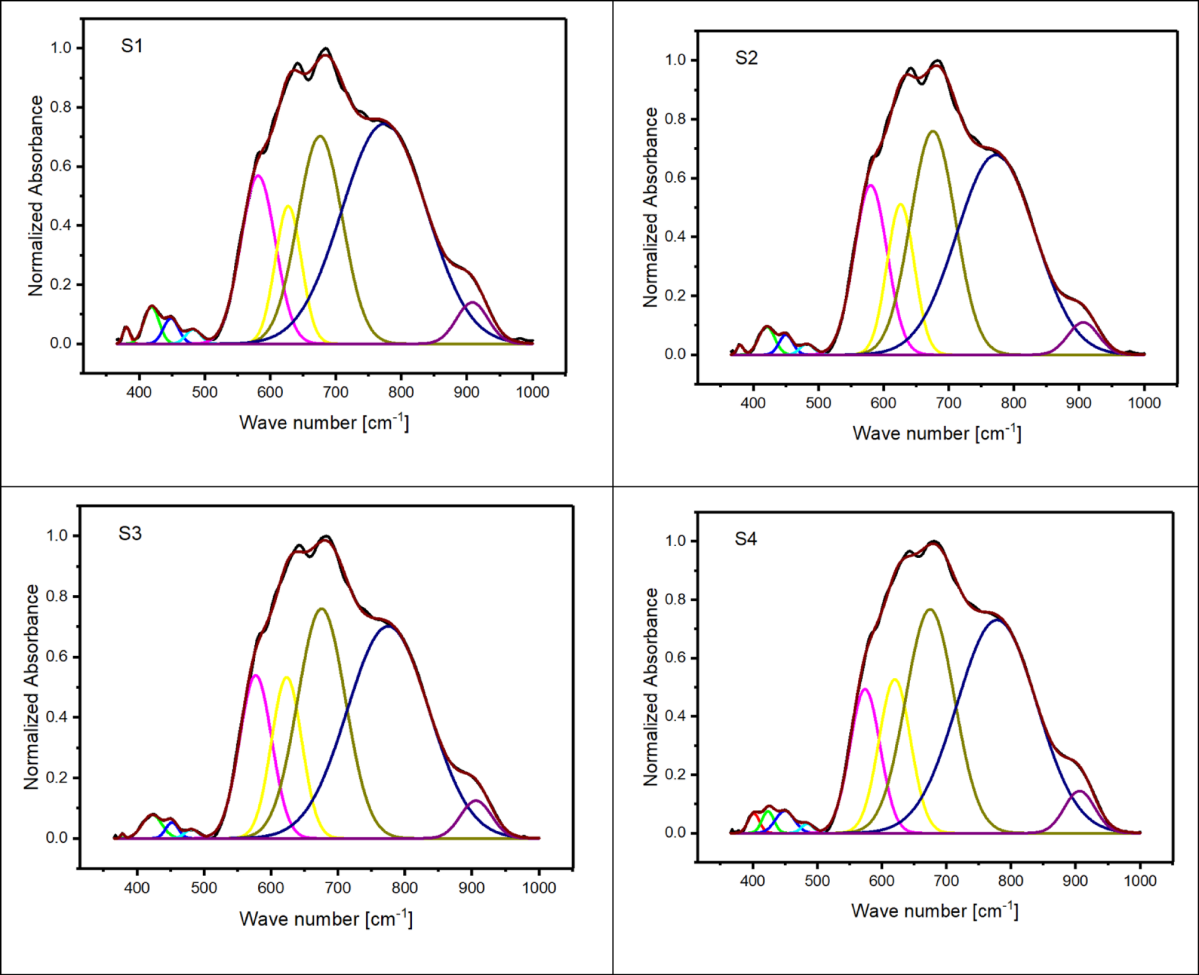

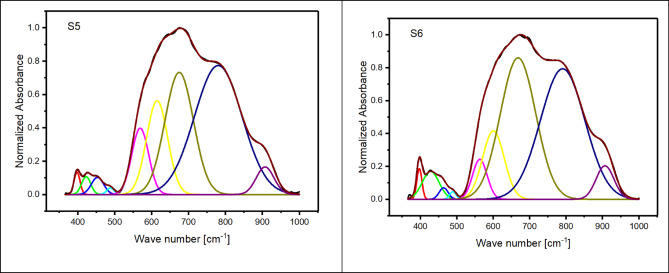
Nine deconvoluted bands of the FTIR spectra for the glass system under investigation from S1 to S6.

 The initial structural analysis of the 60TeO_2_–12.5Nb_2_O_5_–12.5ZnO–(15–*x*)LiF–*x*Pr_2_O_3_ glasses (with *x* ranges from 0.5 to 5.0 mol%) is conducted using FTIR spectroscopy (see Fig. 2). The deconvolution of the spectra, which is presented in Fig. 3, shows nine distinct bands located at approximately 400, 420, 450, 480, 570, 620, 675, 775, and 908 cm^− 1^ (details are provided in Table 2). Based on references^30–48^, these bands are assigned using each component’s relative area (A) and band center (C).

**Table 2:** Deconvolution parameters of FTIR spectra of 60TeO_2_ – 12.5Nb_2_O_5_ – 12.5ZnO – (15 – *x*)LiF – *x*Pr_2_O_3_ glass system. C is the component band center (cm^− 1^) and A is the relative area (%) of the component band.

S1		S2		S3		S4		S5		S6	
C	A	C	A	C	A	C	A	C	A	C	A
380.6	0.296	379.8	0.175	378.3	0.044	---	---	---	---	---	---
---	---	---	---	---	---	401.7	0.576	398.1	0.974	397.3	1.103
418.7	1.409	420.4	1.205	422.5	1.143	423.6	0.660	422.8	1.200	428.2	3.313
448.5	0.966	450.1	0.735	452.3	0.499	449.9	1.065	453.5	1.538	463.9	0.724
482.5	0.534	482.2	0.398	481.4	0.305	484.6	0.282	488.2	0.347	489.3	0.345
581.5	14.575	580.2	14.991	577.1	13.016	574.0	11.029	569.2	8.136	563.3	4.079
627.5	9.018	626.1	10.992	623.5	12.069	620.1	12.364	615.0	14.364	600.6	10.382
675.9	23.138	675.8	26.888	675.5	27.156	674.7	27.620	674.8	25.285	668.3	35.177
774.0	46.805	772.6	41.982	774.9	42.875	778.2	43.151	780.4	44.599	790.7	40.680
908.2	3.257	906.4	2.634	906.1	2.893	906.2	3.254	907.3	3.557	907.0	4.197

Upon adding 0.5 to 2 mol% of Pr_2_​O_3_​ to theTeO_2_–Nb_2_O_5_–ZnO–LiF–Pr_2_O_3_ glass system, the two vibrational bands located at 380 and 420 cm^− 1^ exhibit interesting changes. The 380 cm^− 1^band shifts to a lower frequency, while the 420 cm^− 1^ band shifts to a higher one. Both bands also decrease in area. These initial changes suggest that the vibrations are linked to Te-F-Pr bonds, and the decrease in their areas confirms the decrease in the formation of Non-bridging Fluoride bonds. However, as the Pr_2_​O_3_​concentration increases further (up to 5 mol%), replacing some of the LiF, the changes become more significant. The 380 cm^− 1^band vanishes completely and is replaced by a new band at 400 cm^− 1^. This new band shifts to lower frequencies and grows in area. Simultaneously, the 420 cm^− 1^ band also increases in area. These changes indicate that the vibrations are now attributed to the formation of new Te-O-Pr bonds. The band around 450 cm^− 1^is caused by the presence of Zn^2+^ ions, which are arranged as tetrahedral ZnO_4_ units. The band at 480 cm^− 1^, meanwhile, is assigned to the bending vibrations of Te-O-Te bonds, which overlap with the stretching of Te-O-Nb bonds. The observed decrease in the 480 cm^− 1^ band’s area, along with its shift to higher frequencies, suggests that the Te-O-Te linkages are becoming more rigid and that niobium ions are becoming less localized around tellurium ions. This confirms the creation of continual Te-O-Te and Nb-O-Nb bonds. The intensity and area of the band at approximately 570 cm^− 1^ decrease as Pr_2_O_3_content increases from 0.5 to 5.0 mol%, which suggests less formation of NbO_4_ units. The intensity of the 620 cm^− 1^ band, attributed to the asymmetric axial stretching modes of Te-F bonds in the Te^4+^ tbp network, decreases as the concentration of Pr_2_O_3_ rises from 0.5 to 5.0 mol%. Concurrently, the area of the band at 670 cm^− 1^, attributed to the stretching modes of newly formed Te-O bonds, increases (see Fig. 3; Table 2). This transition confirms that the substitution of LiF with Pr_2_O_3_, which introduces oxygen anions and raises the oxygen-to-tellurium ratio (see Table 1), directly causes the formation of Te-O bonds at the expense of Te-F bonds within the Te^4+^ tbp network. The areas of both the 620 and 670 cm^− 1^ bands increase as the content of Pr_2_O_3_ increases from 0.5 to 5.0 mol%, providing evidence for the enhanced formation of Te^4+^ tbp network bonds.The area of the 770 cm^− 1^ band, which corresponds to the stretching modes of Te^3+^ trigonal pyramids (tp), decreases as Pr_2_O_3_ content rises from 0.5 to 5.0 mol% (see Table 2). This confirms a change from Te^3+^ to Te^4+^ trigonal bipyramids (tbp) groups. This band also shifts to higher frequencies, which indicates increased bond rigidity. Furthermore, the intensity of the 908 cm^− 1^ band, corresponding to NbO_6_ octahedral units, remains largely unchanged. This stability confirms the ongoing presence of NbO_6_ units, which in turn leads to an increase in the cross-link density of the glass network (see Table 3). The cross-link density is calculated according to the bond compression model^49,50^.

**Table 3:** N_4_ values obtained from FTIR and XRD analysis and parameters of cross-link density(n_c_), oxygen molar volume (V_O_), fluoride molar volume (V_F_), oxygen packing density (OPD), and fluoride packing density (FPD) together with their statistical ANOVA results.

Sample code	*N*_4(FTIR)_	*N*_4(XRD)_	*n*_c_	Z_cal_	V_O_ m^3^/ (kg.mol)	Z_cal_	V_F_ m^3^/(kg.mol)	Z_cal_	OPD mol/L	Z_cal_	FPD mol/L	Z_cal_
**S1**	0.407	0.410	2.300	−4.16	0.01533	2.85	0.20770	−2.66	65,248	−2.84	4815	2.97
**S2**	0.474	0.473	2.353	−0.54	0.01531	2.21	0.21651	−2.16	65,322	−2.21	4619	2.23
**S3**	0.478	0.478	2.360	−0.06	0.01527	0.89	0.23614	−1.02	65,477	−0.89	4235	0.78
**S4**	0.481	0.481	2.369	0.53	0.01523	−0.51	0.25898	0.29	65,641	0.50	3861	−0.63
**S5**	0.471	0.473	2.373	0.85	0.01519	−1.96	0.28593	1.84	65,814	1.96	3497	−2.00
**S6**	0.528	0.530	2.410	3.38	0.01515	−3.48	0.31821	3.70	65,994	3.49	3143	−3.34
**Mean**			2.361		0.01525		0.25391		65,583		4028	
**STDEV**			0.036		0.00007		0.04251		289		649	

**Fig. 4:**
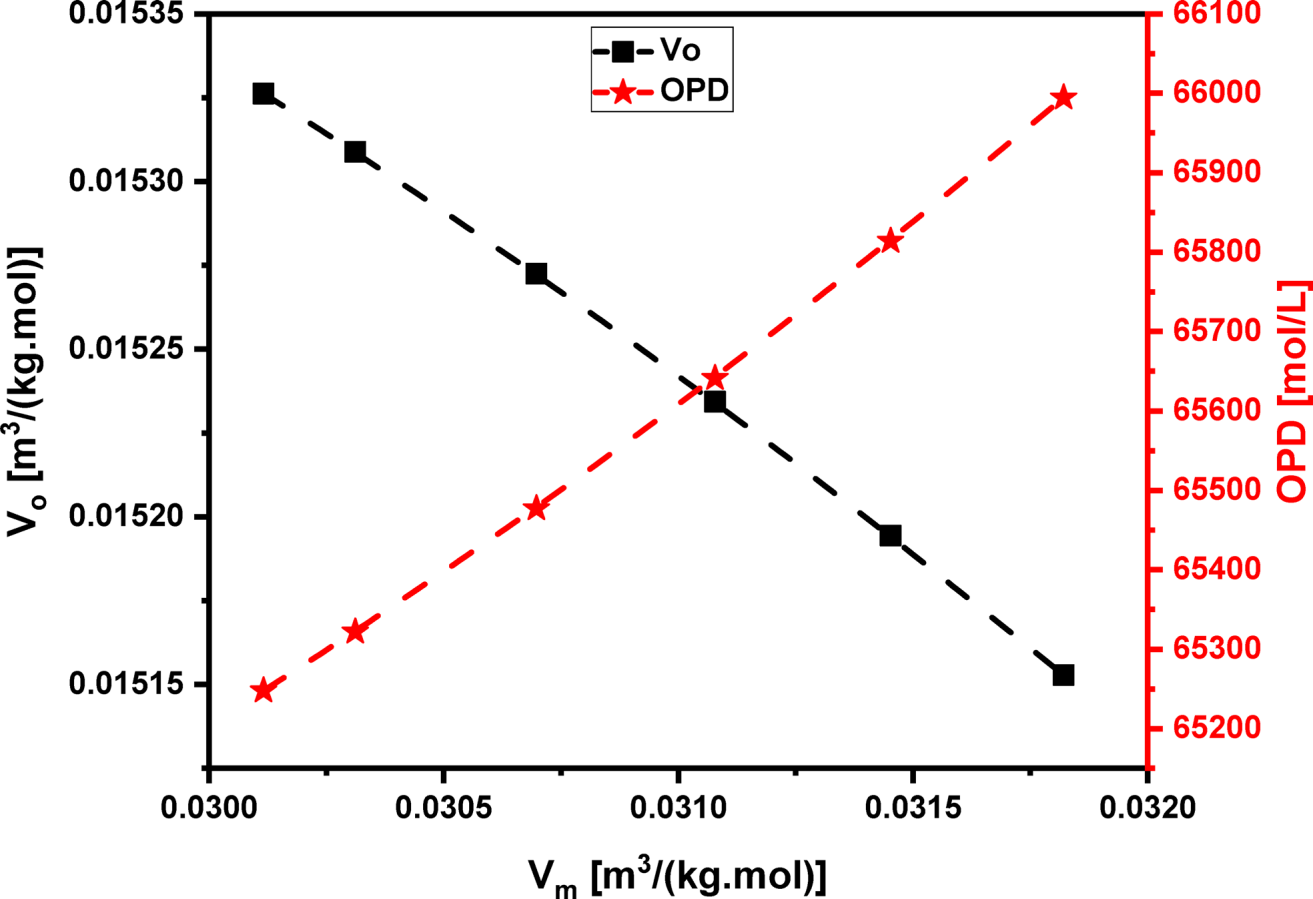
Behaviors of oxygen molar volume (V_O_) and oxygen packing density (OPD) for the glass system under investigation with molar volume (V_m_) as indicator of Pr_2_O_3_ insertion effect.

 Table 3 shows that adding Pr_2_O_3_ from 0.5 to 5.0 mol% increases the fraction of Te^4+^ tbp units (N_4_​) from 0.407 to 0.528, a value calculated from deconvoluted peak intensities. This trend confirms the transformation of Te^3+^ trigonal pyramids (tp) into Te^4+^ trigonal bipyramids (tbp). Further evidence for this structural change comes from changes in the glass’s molar volume and packing density. The decrease in oxygen molar volume (V_o_​) and increase in oxygen packing density (OPD), as seen in Fig. 4; Table 3, suggest a greater formation of continuous bridging oxygen bonds (e.g., Nb-O-Nb, Zn-O-Zn, and Te-O-Te). Conversely, the increase in fluoride molar volume (V_F_​) and decrease in fluoride packing density (FPD), also shown in Table 3, confirm that non-bridging fluoride bonds (e.g., Te-F-Li, Te-F-Zn, Te-F-Nb, and Te-F-Pr) are being formed^51^.These results collectively confirm that substituting LiF with Pr_2_O_3_ increases bridging oxygen bonds at the expense of non-bridging fluoride bonds.

### Gaussian fitting for radial distribution function (RDF)

**Fig. 5:**
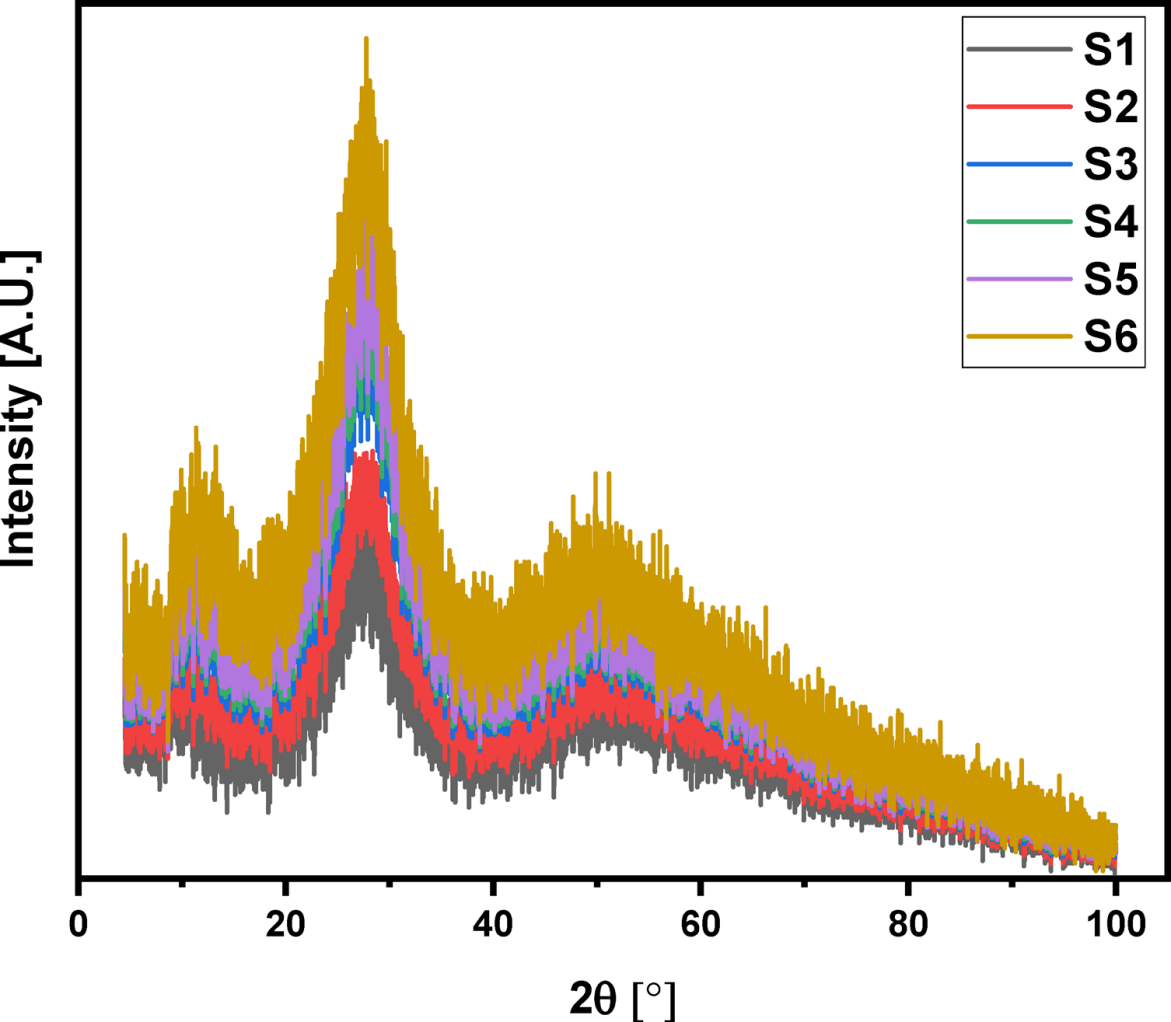
X-ray diffraction patterns (XRD) of 60TeO_2_–12.5Nb_2_O_5_–12.5ZnO–(15 – *x*)LiF–*x*Pr_2_O_3_ glass system in the 2θ range from 4 to 100 degrees.

**Fig. 6:**
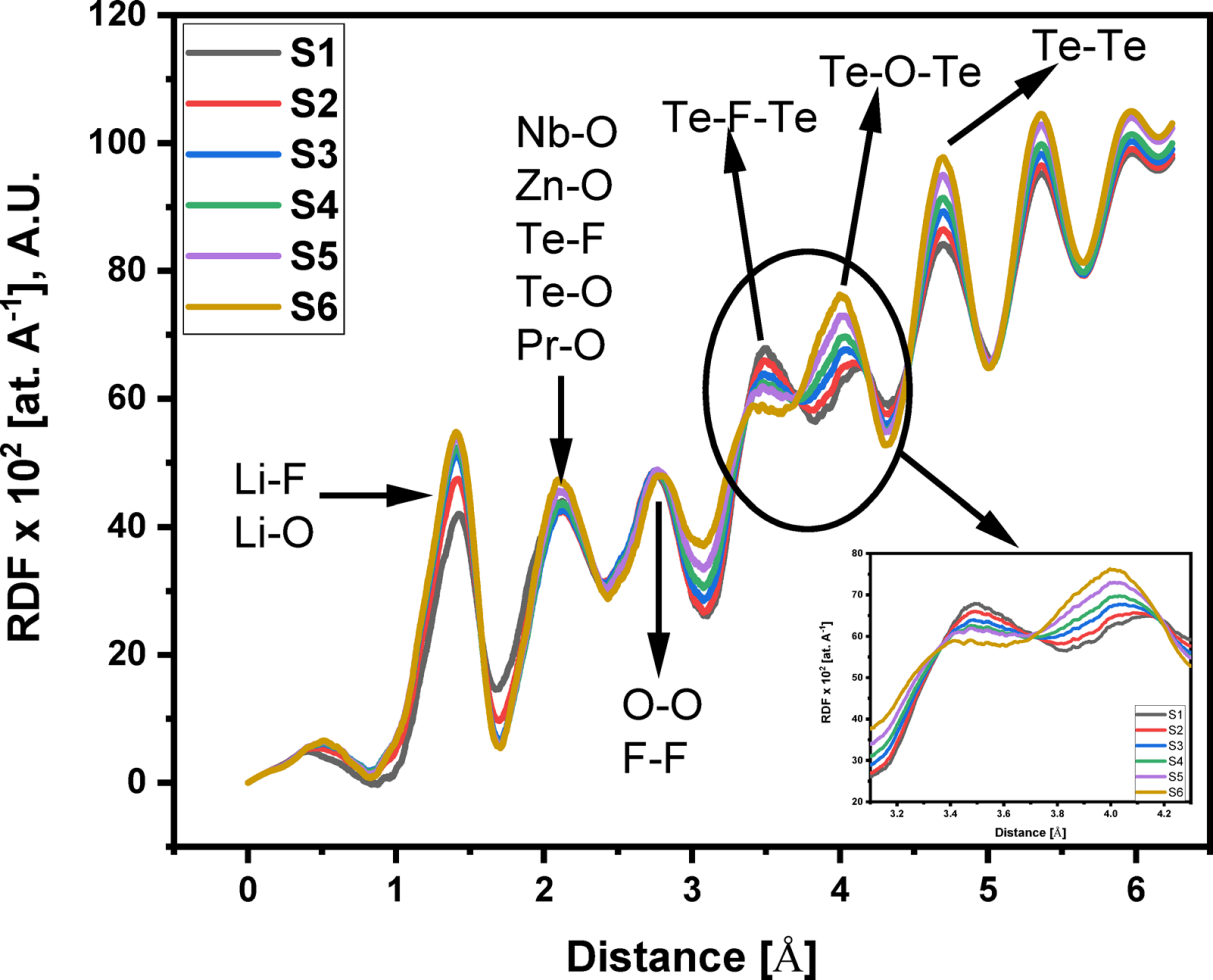
Radial distribution functions (RDF) of the 60TeO_2_–12.5Nb_2_O_5_–12.5ZnO–(15 – *x*)LiF–*x*Pr_2_O_3_ glass system as a function of inter-atomic distance up to 6.5 Å.

**Fig. 7:**
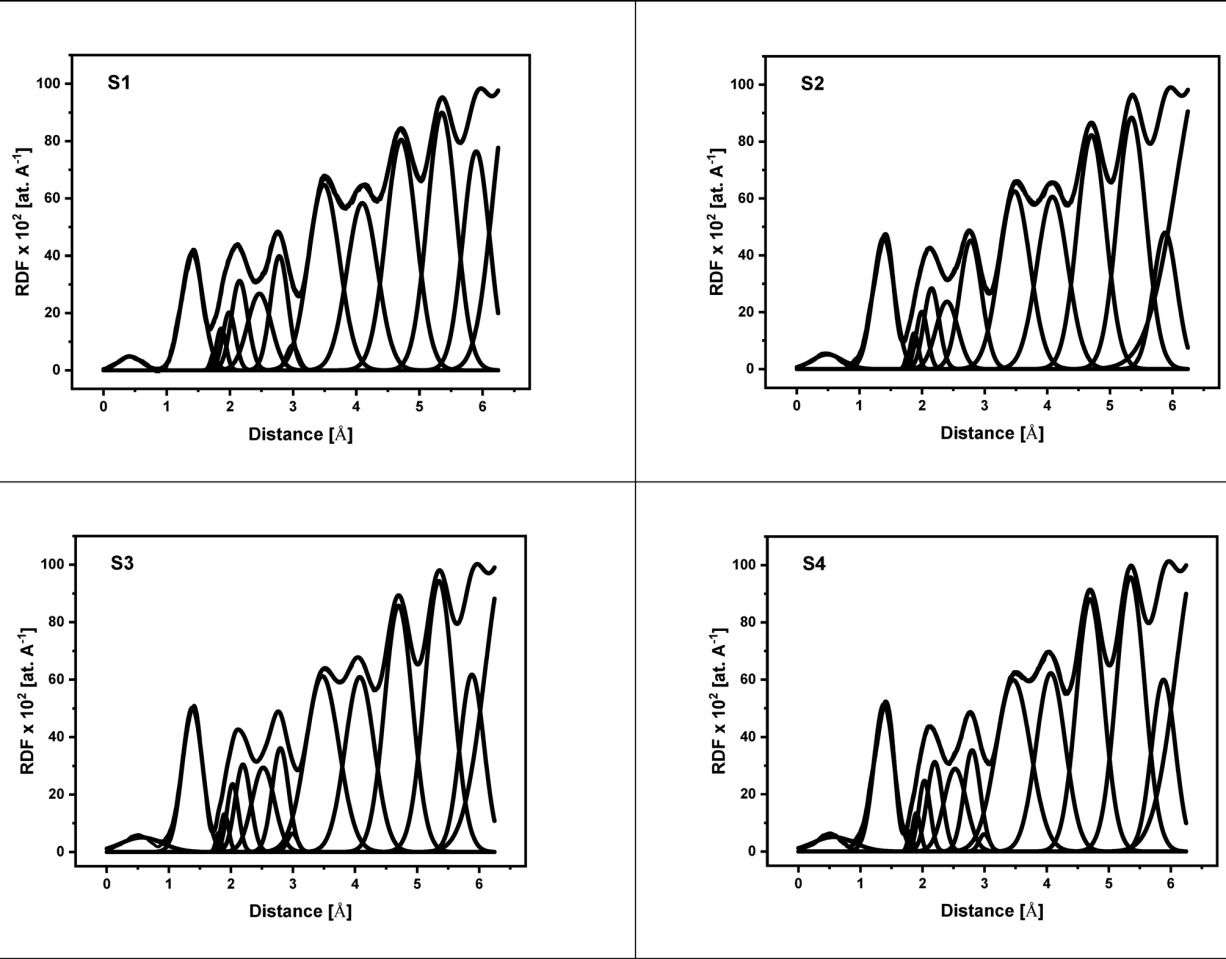

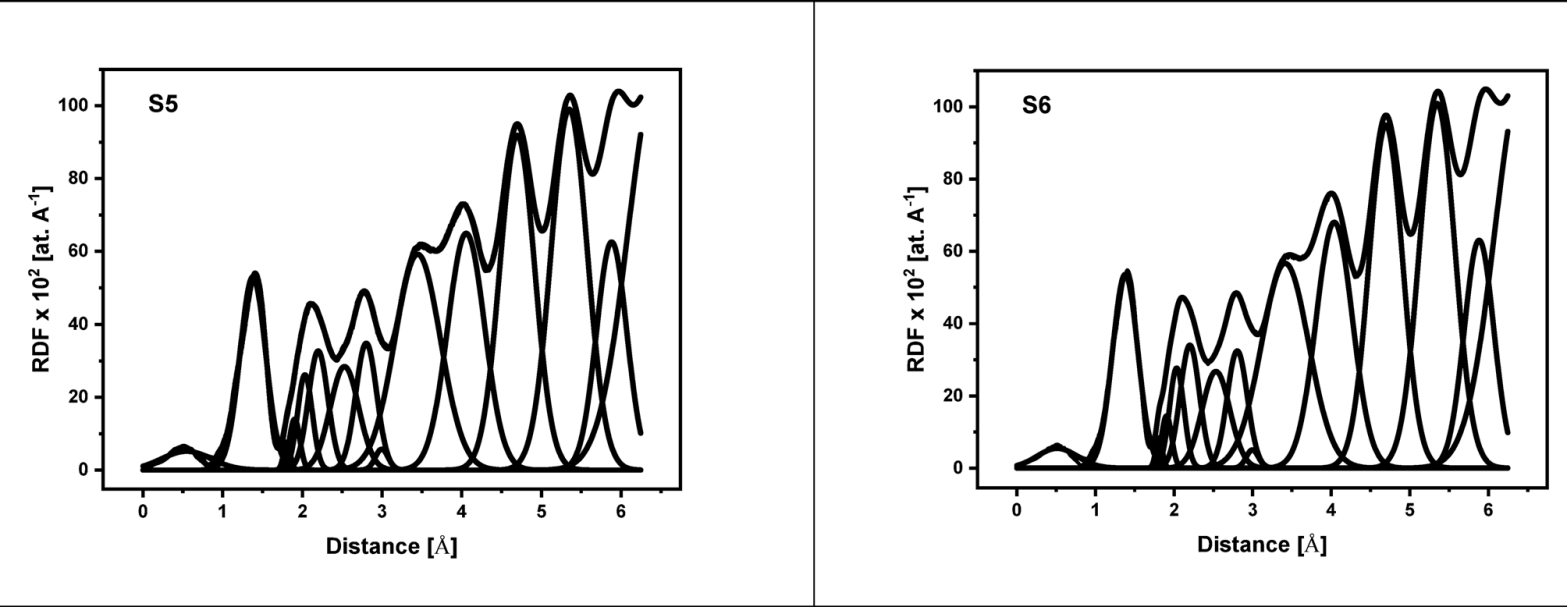
Deconvoluted radial distribution functions (RDF) and Gaussian fitting for the (RDF) curve of each glass sample from S1 to S6.

 The X-ray diffraction (XRD) data for the 60TeO_2_–12.5Nb_2_O_5_–12.5ZnO–(15–*x*)LiF–*x*Pr_2_O_3_ glass system is analyzed to determine its structural parameters. Figures 5 and 6, and 7 display the XRD pattern, radial distribution function, and its corresponding Gaussian curve fitting for the entire glass series. By applying a curve-fitting technique to this XRD data, the structural parameters listed in Table 4 are determined. The analysis of XRD patterns, consistent with previous studies on tellurite glasses^10,11,17,21,52–58^, reveal an initial broad peak at around 1.4 Ǻ. This peak represents nearest-neighbor correlation between lithium and fluoride ions. The second peak at around 2 Ǻ is linked to multiple correlations, including Te-F, Te-O, Nb-O, Zn-O, and Pr-O, indicating the presence of both Te^3+^ trigonal pyramids and Te^4+^ trigonal bipyramid structures. The third peak at about 2.7 Ǻ is attributed to correlations between fluorine and oxygen atoms coordinated with tellurium (F_Te_-F_Te_ and O_Te_-O_Te_). The fourth peak around 3.4 Ǻ, corresponding to the second correlation between tellurium atoms (Te-F-Te), is observed to decrease in height as the lithium fluoride (LiF) content decreases (see Fig. 6). Conversely, the fifth peak at 4 Ǻ, assigned to the second correlation between tellurium and oxygen atoms (Te-O-Te), increases in height as the amount of Pr_2_​O_3_​ increases, replacing fluoride ions with oxygen ions. This change indicates a transition from bridging fluoride bonds to bridging oxygen bonds between tellurium ions, a finding that aligns with the results from FTIR analysis. Based on Figs. 6 and 7 and the corresponding FTIR results, the lithium fluoride (LiF) appears to be structurally distinct from the other components of the material. Specifically, lithium ions interact with other oxides primarily through non-bridging fluoride bonds, while tellurium, niobium, zinc, and praseodymium ions are predominantly connected by bridging oxygen bonds. Additionally, structural parameters, including the coordination number (N_ij_​) and interatomic distance (r_ij_​) between atoms i and j, are calculated using the radial distribution function (RDF) to successfully model the observed RDF curves up to 6.5 Ǻ.

**Table 4:** Structural parameters of 60TeO_2_ – 12.5Nb_2_O_5_ – 12.5ZnO – (15 – *x*)LiF – *x*Pr_2_O_3_ glasses calculated by curve fitting method of X-ray diffraction data. (r) is the inter-atomic distance in Ǻ and (N) is the coordination number.

Interaction	Glass composition						
		S1	S2	S3	S4	S5	S6
**Li-F**	r (Ǻ) N	1.404 5.696	1.386 5.745	1.381 5.713	1.380 5.708	1.380 5.718	1.379 5.726
**Nb-O**	r (Ǻ) N	1.745 5.769	1.781 5.818	1.801 5.820	1.804 5.826	1.806 5.832	1.808 5.838
**Zn-O**	r (Ǻ) N	1.862 3.919	1.882 3.911	1.898 3.916	1.902 3.915	1.903 3.914	1.905 3.913
**Te-F**	r (Ǻ) N	1.988 1.142	2.012 1.223	2.029 1.258	2.031 1.297	2.032 1.302	2.033 1.332
**Te-O**	r (Ǻ) N	2.154 2.268	2.177 2.250	2.194 2.193	2.197 2.184	2.198 2.171	2.201 2.198
**Pr-O**	r (Ǻ) N	2.468 5.446	2.503 5.448	2.521 5.452	2.525 5.439	2.528 5.435	2.533 5.431

**Fig. 8:**
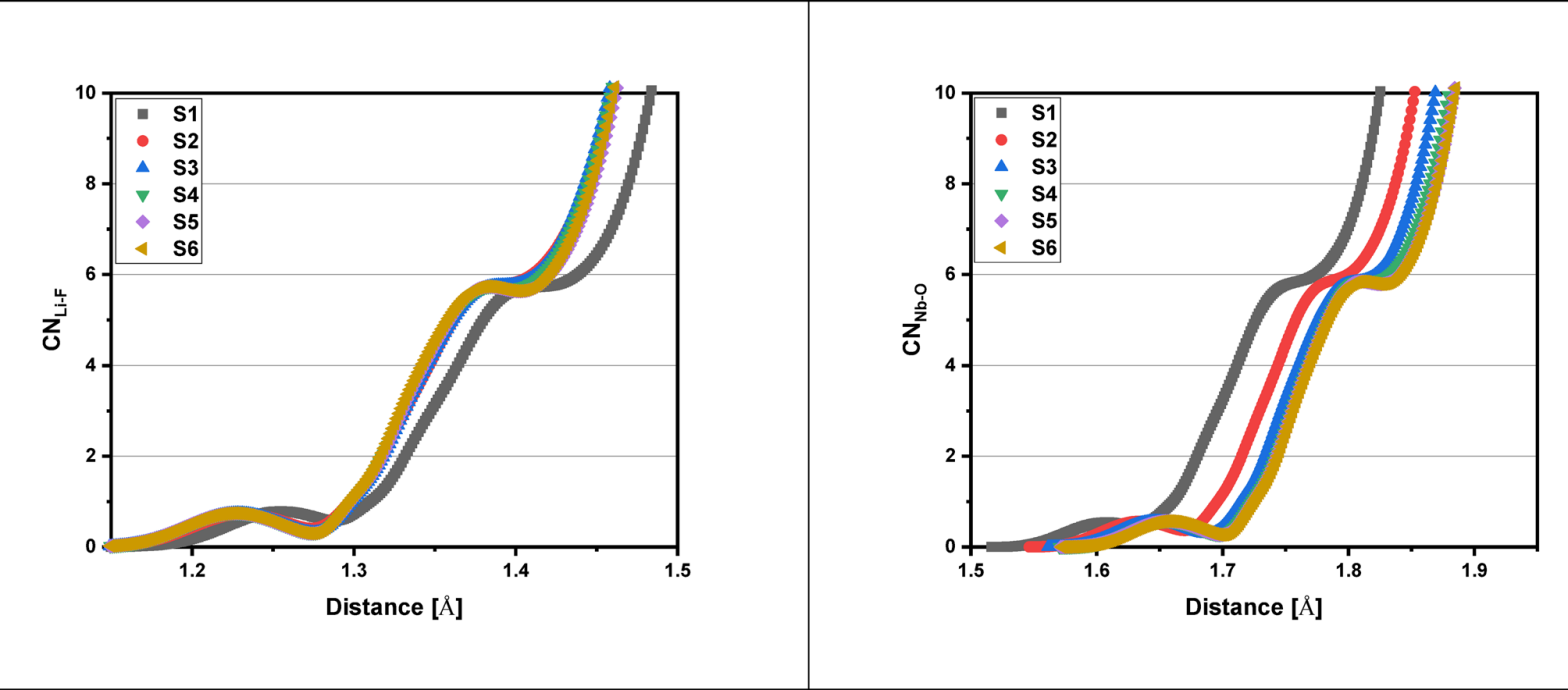

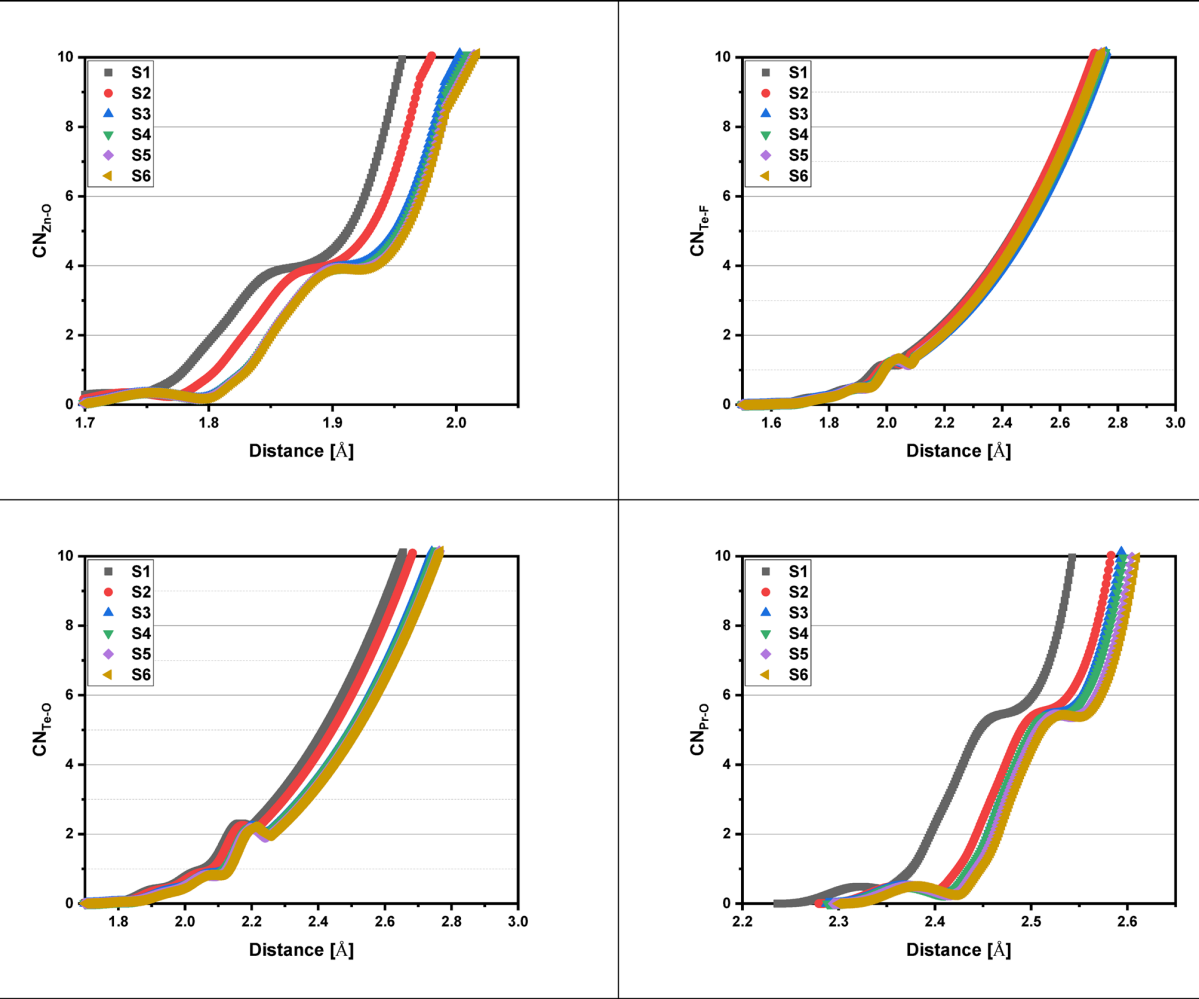
Coordination number (CN) curves of each constituent cation in all glass samples as a function of inter-atomic distances.

 Each praseodymium atom is surrounded by six oxygen atoms at approximately 2.5 Å, affirming its six-coordinated state. This structural arrangement is further supported by the coordination number curves for all ions (Pr^3+^,Te^4+^, Nb^3+^, Zn^2+^, and Li^2+^) shown in Fig. 8 and summarized in Table 4, which are based on calculations from established references^59–63^. With the addition of praseodymium oxide (from 0.5 to 5.0 mol %), key structural transformations occur. The niobium ions’ mean coordination number increases from 5.769 to 5.838, confirming a shift from NbO_4_ to NbO_6_ units. Similarly, the tellurium atoms’ mean coordination number increases from 3.410 to 3.530. This change, which is also supported by FTIR studies and N_4_ values from FTIR and XRD data in Table 3, confirms the transformation from trigonal pyramidal (TeO_3_) units to trigonal bipyramidal (TeO_4_) units. In this context, each tellurium atom is predominantly coordinated with two oxygen ions and one fluoride ion at approximately 2.0 Å, and the number of surrounding fluoride ions increases with a greater presence of non-bridging fluoride ions. The mean coordination number of lithium ions also shows a slight increase from 5.696 to 5.745, confirming their six-coordinated state. Conversely, zinc atoms consistently maintain a coordination number of four, as they are surrounded by four oxygen ions at approximately 1.9 Å, and this value remains constant across all glass compositions.

Replacing LiF with Pr₂O₃, results a more oxygen atoms that are added instead of fluorine atoms, which replace the fluoride links in the network. Results of RDF and FTIR investigations showed the change in type of bonding from Te–F–Pr to Te–O–Pr, and consequently the number of TeO_4_ (trigonal bipyramids, (tbp)) units increases. The coordination numbers for Te and Nb increases, which indicates that the network comes up stronger and the cross-linking density increases.

### DSC and glass transition temperature

**Fig. 9:**
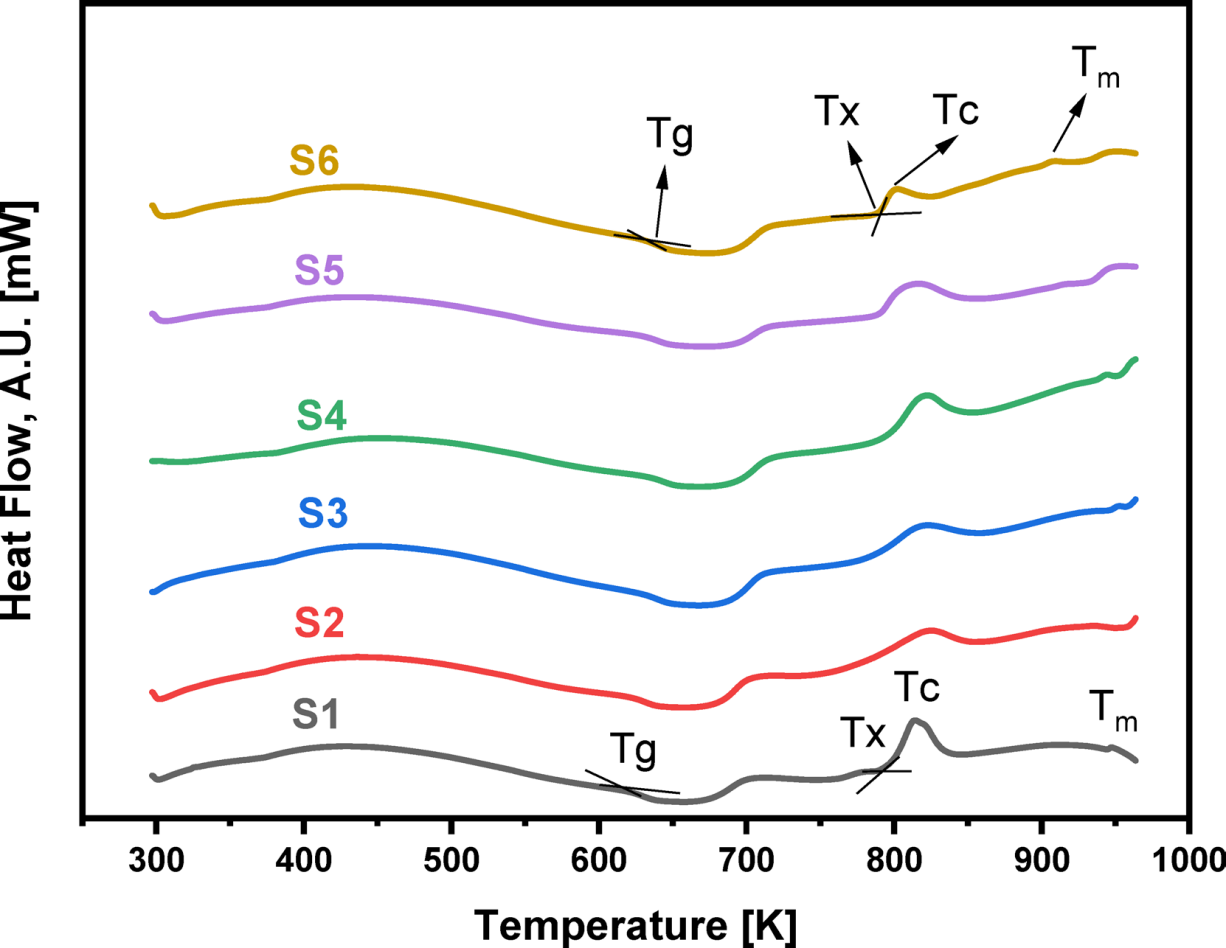
DSC curves of the 60TeO_2_–12.5Nb_2_O_5_–12.5ZnO–(15 – *x*)LiF–*x*Pr_2_O_3_ glass system in the temperature range of 297 to 964 K.

**Fig. 10:**
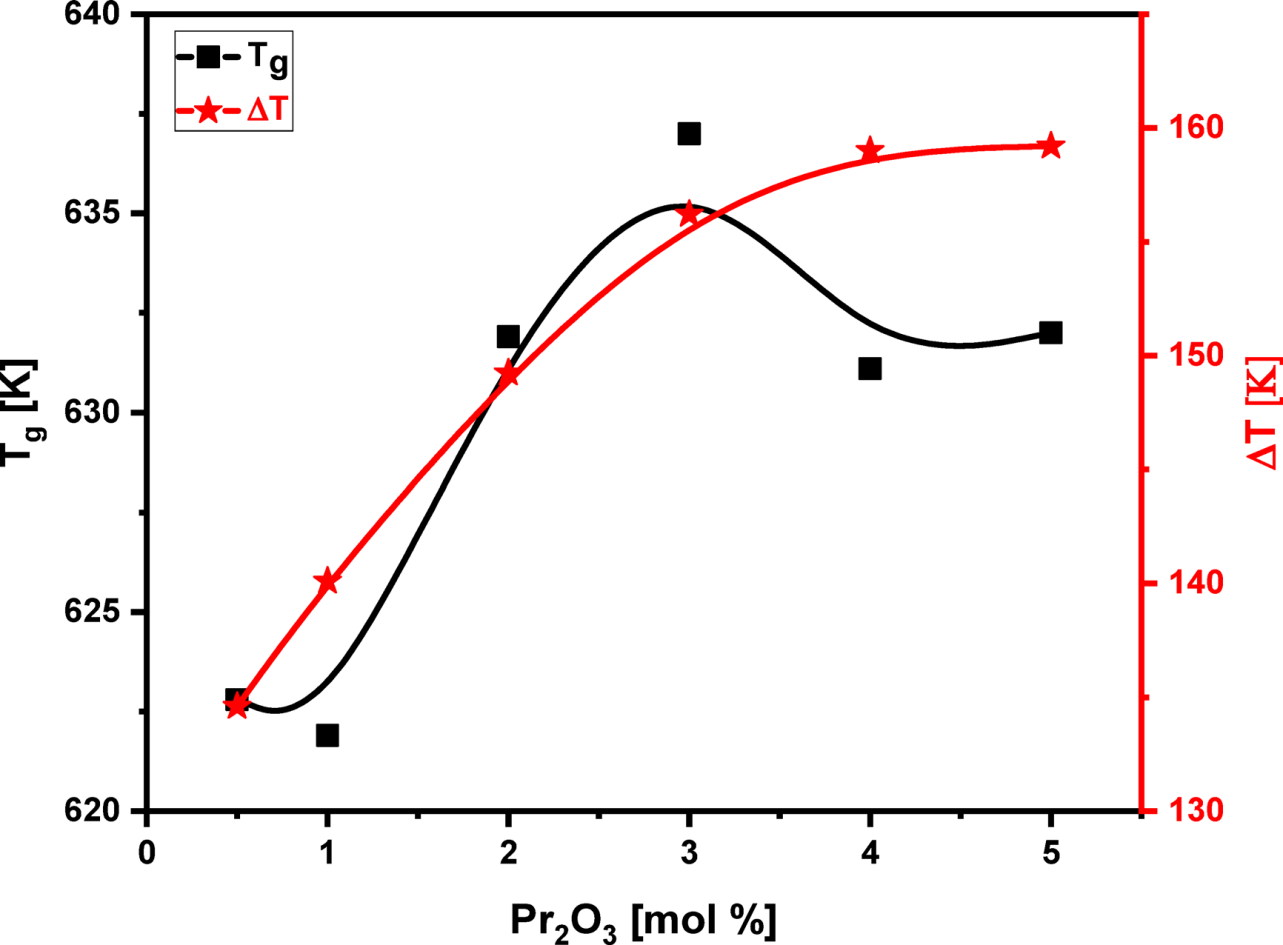
Variation of glass transition temperature (T_g_) and thermal stability (ΔT) for the glass system under investigation with Pr_2_O_3_ content from 0.5 to 5.0 mol%.

 Adding Pr_2_O_3_ to the glass network while replacing LiF results in an increase in the glass transition temperature (T_g_​) from 622.8 to 632.0 K, as shown in the DSC curves in Figs. 9 and 10. This rise in T_g_​ is a direct indicator of increased fractions of TeO_4_ (trigonal bipyramids, (tbp)) units, cross-link density, and rigidity within the glass network structure. This effect is attributed to changes in the coordination of network-forming atoms and the creation of more strong bridging oxygen bonds^64^, a conclusion supported by FTIR and RDF results. Consistent with the increase in T_g_, the onset crystallization temperature (T_x_​) also rises from 757.4 to 791.2 K.The glass’s thermal stability is measured by the glass criterion factor ΔT = T_x_​−T_g_​^65^. A larger ΔT (higher than 100 K) indicates a stronger resistance to crystallization. In this case, ΔT increases from 134.6 K to 159.2 K, see Table 5, as Pr_2_O_3_ replaces LiF from 0.5 to 5.0 mol%, demonstrating an improvement in the glass’s thermal stability. The thermal analysis results show that sample S6 has the best thermal stability, and the mixed glass-former samples possess superior anti-crystallization abilities compared to pure tellurite glass (ΔT = 80 K)^66^.The increased rigidity of the glass is confirmed by the rise in both thermal stability (ΔT) and glass-forming ability (T_rg_​=T_g_​/T_m_​) as Pr_2_O_3_ content rises from 0.5 to 5.0 mol% ^65^. The T_rg_​ values, which increase from 0.519 to 0.564, fall within the typical range of 1/2 to 2/3, as proposed by the Kauzmann hypothesis^67,68^, further supporting the enhanced stability due to the increased TeO_4_ tbp units that leads to the increased cross link density, and the increased strong bridging oxygen bonds as reached by FTIR and RDF results. Table 5 provides a detailed summary of these thermal properties, including the glass transition temperature (T_g_), onset crystallization temperature (T_x_​), crystallization temperature (T_c_​), melting temperature (T_m_​), thermal stability (ΔT), and glass forming ability (T_rg_​).

**Table 5:** Glass transition temperatures (T_g_), onset crystallization temperature (T_x_), melting temperature (T_m_), thermal stability (ΔT), and glass forming ability (T_rg_).

Sample code	T_g_ K	T_x_ K	T_c_ K	T_m_ K	ΔT K	T_rg_
**S1**	622.8	757.4	814.2	947.4	134.6	0.519
**S2**	621.9	762.0	825.4	936.1	140.1	0.526
**S3**	631.9	781.1	822.8	952.9	149.2	0.528
**S4**	637.0	793.2	822.7	944.6	156.2	0.542
**S5**	631.1	790.1	816.0	915.5	159.0	0.557
**S6**	632.0	791.2	802.4	909.6	159.2	0.564

### Density and molar volume

**Fig. 11:**
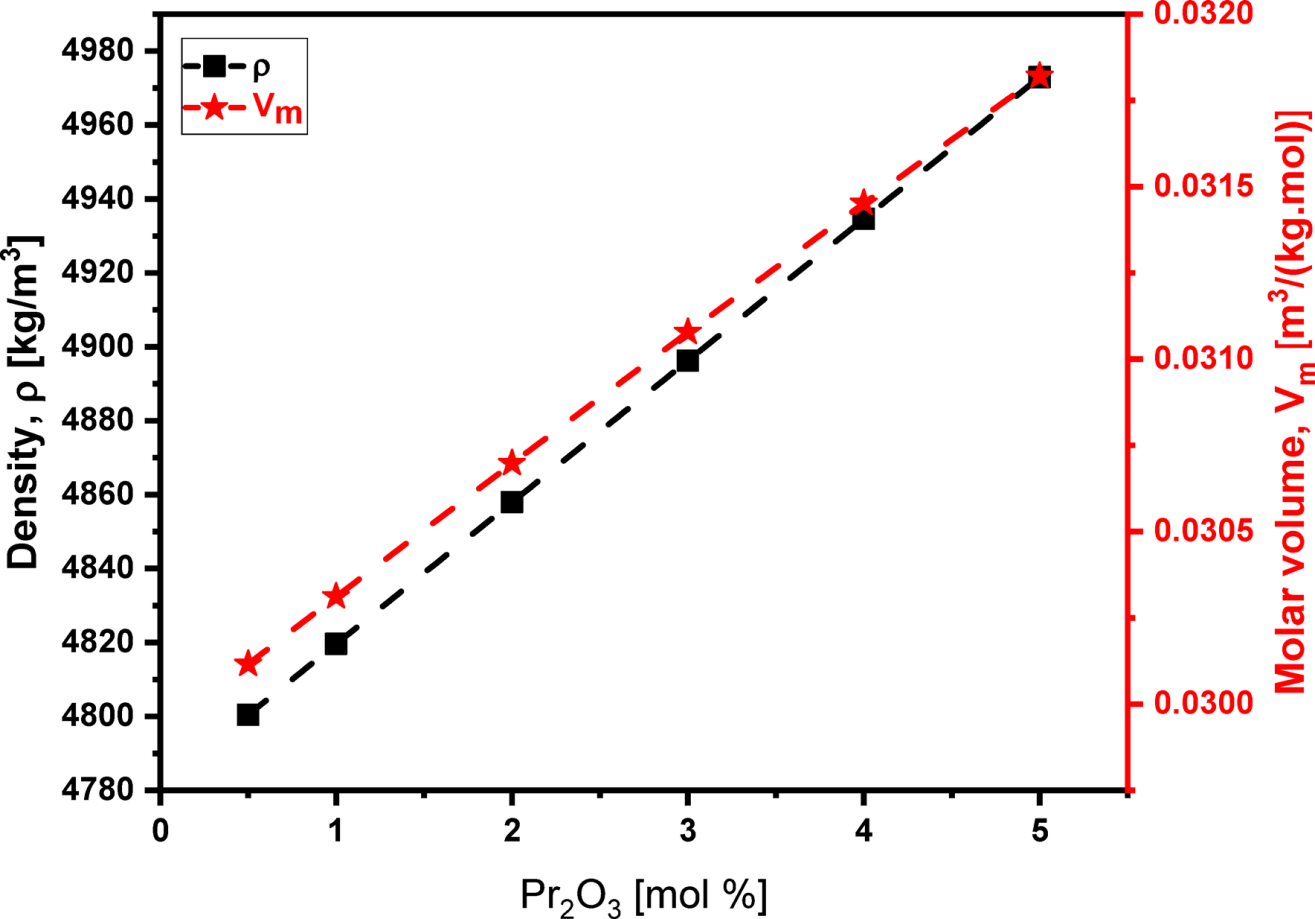
Behaviors of both density and molar volume for the glass system under investigation with Pr_2_O_3_ content from 0.5 to 5.0 mol%.

 The experimental values of density (ρ), molar volume (V_m_), oxygen molar volume (V_O_), fluoride molar volume (V_F_), oxygen packing density (OPD), and fluoride packing density (FPD) of the studied glass system are presented in Tables 1 and 3. Figure 11 illustrates how density and molar volume change with the Pr_2_O_3_ content. As the concentration of Pr_2_O_3_ rises from 0.5 to 5.0 mol%, replacing the lighter LiF, the glass’s density rises from 4800 to 4973 kg/m^3^. This is because Pr_2_O_3_​ has a much higher molecular weight (329.80 g/mol) than LiF (25.94 g/mol). Simultaneously, as the Pr_2_O_3_​ increases, the molar volume –the volume occupied by one gram-mole of the glass– rises from 0.0301 to 0.0318 m^3^/kg.mol. This increase in molar volume is likely a result of two factors. First, the substitution of LiF, which has a smaller molar volume (0.0098 m^3^/kg.mol), with Pr_2_O_3_​​, which has a larger molar volume (0.0478 m^3^/kg.mol). Second, as the Pr_2_O_3_ ​​content increases, there is a rise in the number of non-bridging fluorides (NBFs) within the tellurite network, as indicated by FTIR results. This structural change is further supported by the increase in fluoride molar volume (V_F_​) from 0.2077 to 0.3182 mol/L and the corresponding decrease in fluoride packing density (FPD) from 7814.7 to 3142.6 mol/L^51^^,^^64^^,^^69^.

### Ultrasonic velocities and elastic moduli

**Fig. 12:**
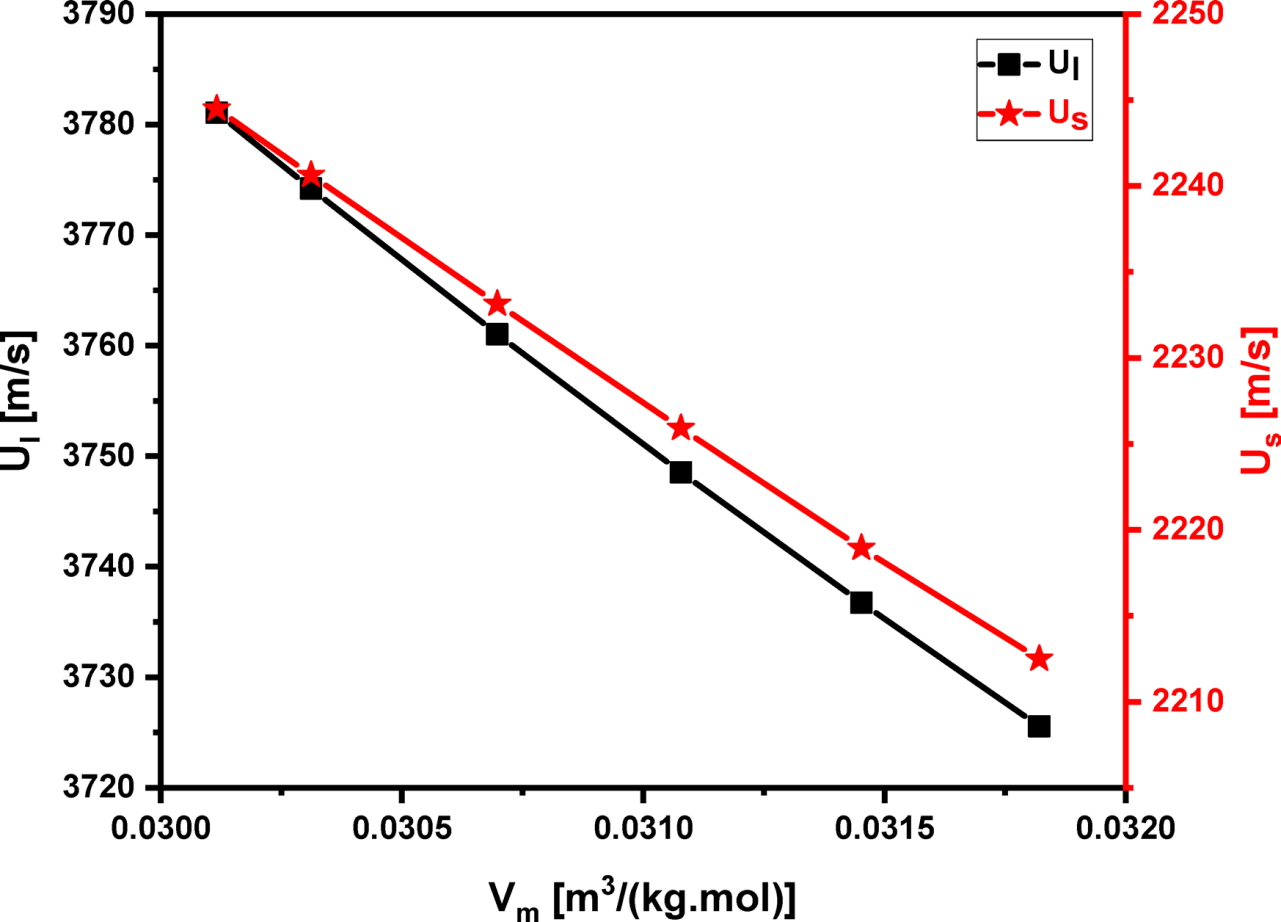
Longitudinal and shear ultrasonic velocities behaviors of 60TeO_2_–12.5Nb_2_O_5_–12.5ZnO –(15 – *x*)LiF–*x*Pr_2_O_3_ glass system with its molar volume.

 As depicted in Fig. 12, the ultrasonic velocities, both longitudinal (U_l_​) and shear (U_s_​), decrease as the mol% content of Pr_2_O_3_ increases in the glass system. The maximum velocities, 3781 m/s for longitudinal waves and 2244 m/s for shear waves, occur at a Pr_2_O_3_ concentration of 0.5 mol%, as detailed in Table 6. Subsequently, increasing the Pr_2_O_3_ content to 5.0 mol% decreases these velocities to 3726 m/s and 2213 m/s, respectively. The general decrease in ultrasonic velocity is attributed to several factors, including an increase in molar volume, a rise in the number of non-bridging fluoride anions (NBFs), and an increase in the average distance between atoms. This last point is due to shorter Li–F bonds being replaced by longer Pr–O bonds. The velocity decrease is also linked to an increase in bridging oxygens, specifically those forming Nb–O–Nb, Zn–O–Zn, and Te–O–Te networks, which is confirmed by FTIR and RDF analysis. This structural change reduces the number of bonds per unit volume (n_b_​). According to the bond compression model^49,50^, n_b_​ declines from 8.219 × 10^28^ to 7.913 × 10^28^ m^− 3^ as the Pr_2_O_3_ content rises from 0.5 to 5.0 mol %, as shown in Table 7.

**Table 6:** Longitudinal ultrasonic velocity (U_l_), shear ultrasonic velocity (U_s_), longitudinal modulus (L), shear modulus (G), Young’s modulus (E), bulk modulus (K), and average atomic ring size (ℓ), together with their statistical ANOVA results.

Sample code	U_l_ m/s	Z_cal_	U_s_ m/s	Z_cal_	L GPa	Z_cal_	G GPa	Z_cal_	E GPa	Z_cal_	K GPa	Z_cal_	ℓ nm	Z_cal_
**S1**	3781	3.02	2244	2.99	68.63	−2.53	24.18	−2.70	59.39	−2.65	36.39	−2.34	5.387	3.21
**S2**	3774	2.24	2241	2.24	68.65	−2.15	24.20	−2.17	59.42	−2.16	36.39	−2.13	5.355	2.17
**S3**	3761	0.74	2233	0.76	68.72	−1.14	24.23	−0.99	59.49	−1.04	36.42	−1.30	5.303	0.51
**S4**	3749	−0.68	2226	−0.66	68.80	0.20	24.26	0.34	59.57	0.30	36.45	0.04	5.264	−0.73
**S5**	3737	−2.02	2219	−2.03	68.90	1.84	24.30	1.82	59.66	1.83	36.51	1.87	5.227	−1.91
**S6**	3726	−3.29	2213	−3.30	69.02	3.77	24.34	3.69	59.77	3.72	36.57	3.85	5.185	−3.24
**Mean**	3755		2229		68.79		24.25		59.55		36.5		36.45	
**STDEV**	22		12		0.15		0.06		0.14		0.1		0.07	

**Table 7:** Mean ultrasonic velocity (U_m_), softening temperature (T_s_), micro-hardness (H), number of bonds per unit volume (n_b_), fractal bond connectivity (D), and the power (α), together with their statistical ANOVA results.

Sample code	U_m_ m/s	Z_cal_	T_s_ K	Z_cal_	H GPa	Z_cal_	*n*_b_×10^28^ m^− 3^	Z_cal_	D GPa	Z_cal_	α	Z_cal_
**S1**	2486	3.00	873	−2.94	4.386	−2.87	8.219	2.18	2.6586	−3.62	1.026	−2.91
**S2**	2481	2.24	875	−2.22	4.390	−2.18	8.262	2.92	2.6596	−2.13	1.028	−2.22
**S3**	2473	0.76	879	−0.80	4.397	−0.82	8.152	1.05	2.6611	0.06	1.032	−0.83
**S4**	2465	−0.66	883	0.60	4.404	0.50	8.050	−0.67	2.6619	1.27	1.036	0.57
**S5**	2457	−2.03	887	1.97	4.411	1.79	7.943	−2.48	2.6621	1.54	1.040	1.99
**S6**	2450	−3.30	891	3.39	4.421	3.59	7.913	−3.00	2.6630	2.89	1.044	3.40
**Mean**	2469		882		4.402		8.090		2.6610		1.034	
**STDEV**	14		7		0.013		0.145		0.0017		0.007	

**Fig. 13:**
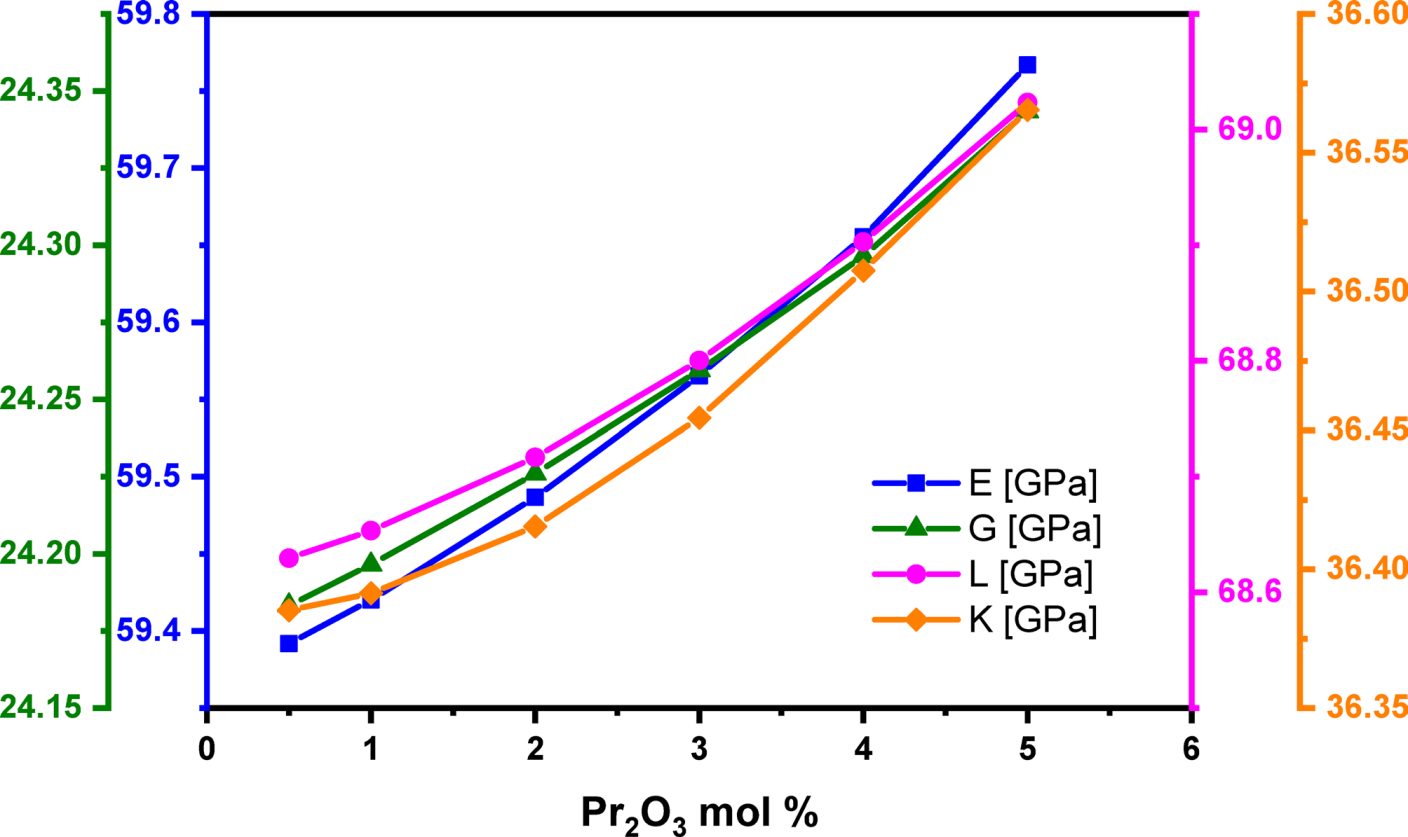
Behaviors of elastic moduli (Youngs, (E), Shear, (G), Longitudinal, (L), and Bulk, (K)) of the glass system with Pr_2_O_3_ content from 0.5 to 5.0 mol%.

 According to Table 6; Fig. 13, the experimentally determined elastic moduli – longitudinal (L), shear (G), Young’s (E), and bulk (K) – all increase slightly as the Pr_2_O_3_ content rises from 0.5 to 5.0 mol%. The values for these moduli rise from 68.63, 24.18, 36.39, and 59.39 GPa to 69.02, 24.34, 36.57, and 59.77 GPa, respectively, with this increase occurring in two distinct phases. This behavior is attributed to several structural changes. The primary cause is the substitution of LiF with Pr_2_O_3_, which replaces one fluoride anion with three oxygen anions. This replacement increases the cross-link density (n_c_​) from 2.300 to 2.410 (as calculated by the bond compression model^49,50^. Another reason is the transformation from trigonal pyramidal (TeO_3_) units to trigonal bipyramidal (TeO_4_) units, with strong bridging oxygen bonds, that led to the increased fractions of N_4_ values. Furthermore, the substitution of LiF (with a bond energy of 577 kJ/mol^70^ with Pr_2_O_3_ (with a higher bond energy of 753 kJ/mol^70^ significantly influences the average bond dissociation energy (G_t_​), which increases from 57.71 to 58.12 kJ/mol (per the Makishima and Mackenzie model^71,72^, as seen in Table 1.

Other contributing factors include a decrease in the average atomic ring diameter (ℓ) from 5.322 to 5.099 nm. Bridge et al. ^63^ proposed a model to determine if the elastic moduli of oxide glasses are explained by their ring diameters. Their reasoning began by noting that the macroscopic elastic deformation of materials, such as the central depression of a loaded beam, scales strongly with its length (proportional to ℓ^4^). They applied this concept to the puckered, non-planar atomic rings, defining their size (ℓ) as the extremal diameter (perimeter divided by *n*). This led to the central hypothesis that the bulk modulus K is generally proportional to f_b_ ℓ^−n^, where f_b_ is the bond bending force constant (assumed proportional to the bond stretching force constant, f). Consequently, by rearranging Eq. (1) and inputting the experimentally determined K, the atomic ring size (ℓ) could be calculated. This model was successfully tested by El-Mallawany et al^73^., who found a ring diameter of 5.0 nm for pure TeO_2_ glass. The atomic ring size (ℓ) has been calculated by using the ring distortion model, Eq. (1).

1 $$\:\text{K}=\frac{0.0106{\text{F}}_{\text{b}}}{{\mathcal{l}}^{3.84}}$$

where K, is the experimental bulk modulus, and F_b_, is the bond bending force. The mean atomic ring size is found to decrease due to the incorporation of lithium and niobium ions creating more octahedral sites in the glass network. This structural change aligns with the shifts in the mean coordination number of tellurium ions, a conclusion supported by both FTIR and XRD results. Collectively, these changes in N_4_, cross-link density, bond strength, and atomic spacing are the key factors determining the glass’s increased rigidity.

The rigidity of the glass structure is further indicated by its softening temperature (T_s_​) and micro-hardness (H), as detailed in Table 7. Both values increase as the Pr_2_O_3_ content increases from 0.5 to 5.0 mol%. Specifically, T_s_​ increases from 873 to 891 K, and H increases from 4.386 to 4.421 GPa. This overall increase in rigidity, including the elastic moduli, is a direct result of structural changes caused by the addition of Pr_2_O_3_. The substitution of LiF (a modifier with weaker Li–F bonds) with Pr_2_O_3_ (which introduces stronger Pr–O bonds) increases the number of strong bridging oxygens that leads to the increased N_4_ values, and consequently a more rigid network. This is supported by an increase in cross-link density (n_c_) from 2.300 to 2.410 and a rise in fractal bond connectivity (D = 4G/K^74^,) from 2.659 to 2.663, as detailed in Tables 3 and 7.

A direct relationship exists between the molar volume (V_m_​) and experimental bulk modulus (K) for ionic solids with similar structures. This relationship, established in earlier research^75^, assumes the Born potential of ionic solids for internal energy (U). As such, the relationship between K_exp_ and V_m_​ can be described using the following equations:

2 $$\:K={V}_{m}\frac{{\partial\:}^{2}U}{\partial\:{V}_{m}^{2}}\:$$

3 $$\:K={V}_{m}^{-\alpha\:}$$

**Fig. 14:**
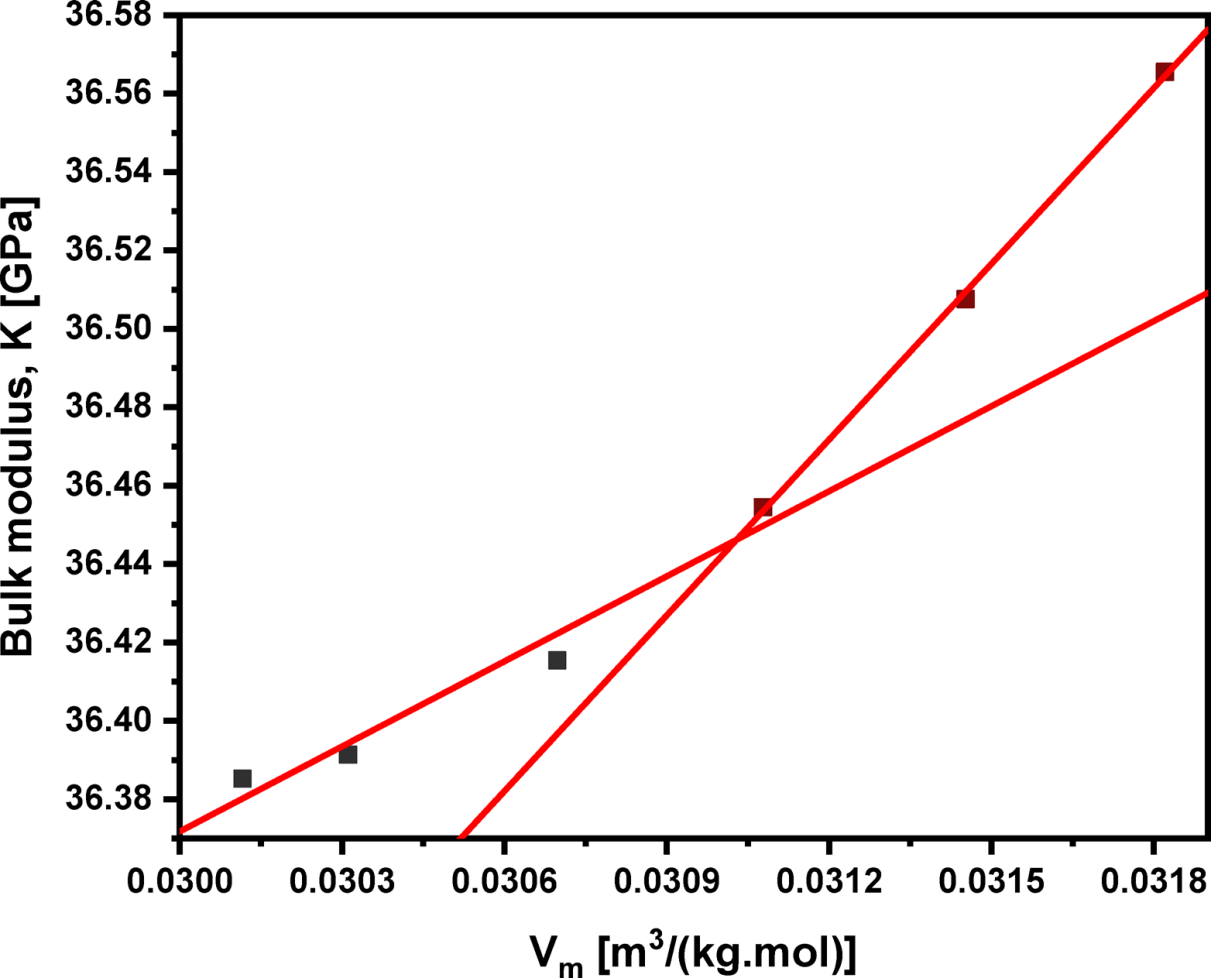
Behavior of experimental bulk modulus of the glass system under investigation with molar volume.

**Fig. 15:**
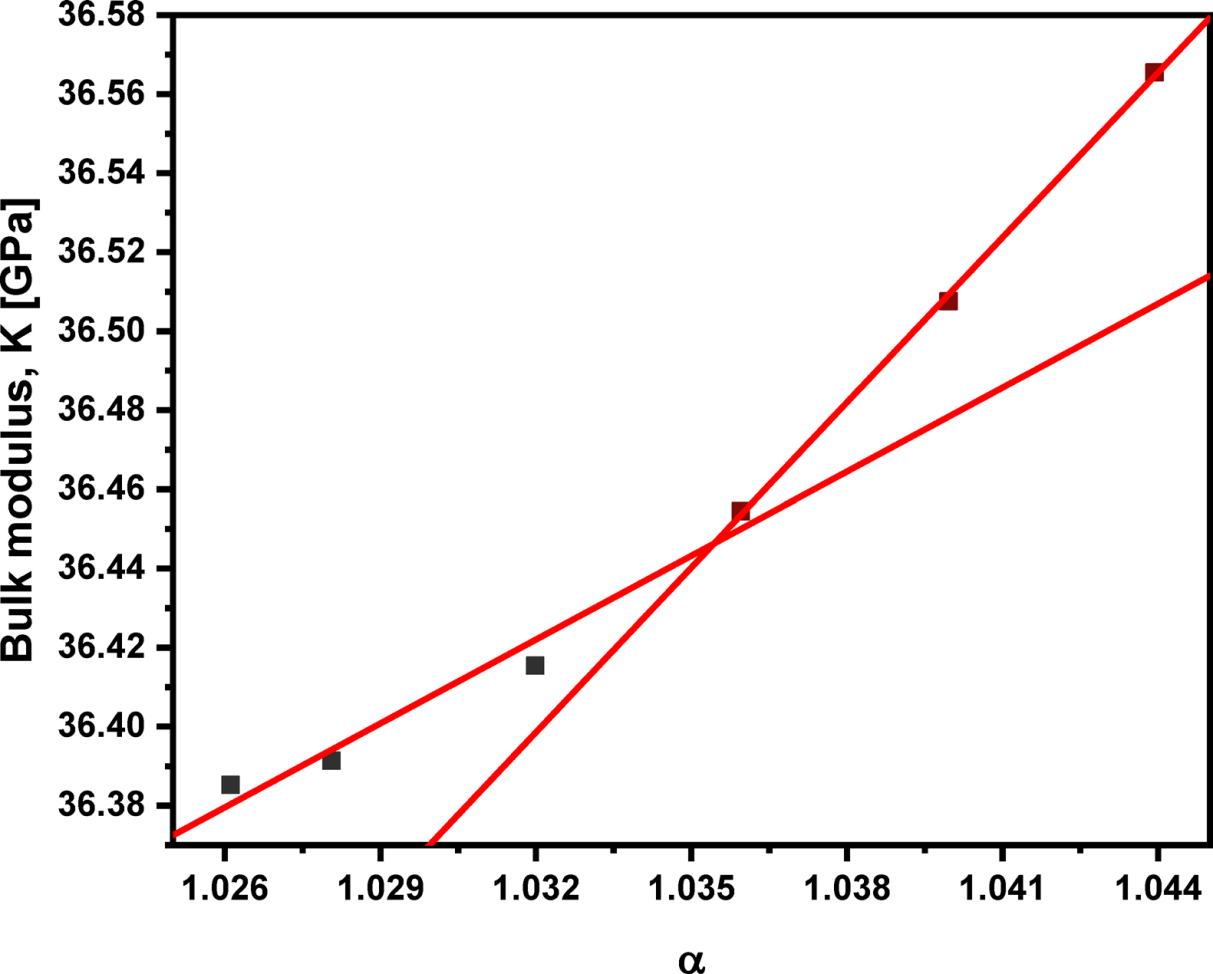
Relationship between the experimental bulk modulus (K) and the power (α) of 60TeO_2_–12.5Nb_2_O_5_–12.5ZnO–(15 – *x*)LiF–*x*Pr_2_O_3_ glass system.

The variation of (α) in the bulk modulus–volume relationships is determined by: (1) the nature of the bonding and (2) the nature of the coordination polyhedra. When the volume changes occur without change in the nature of the bonding or of the coordination plyhedra, log (K) – log (V_m_) plots generally are linear and possess a slope of (− 4/3) ^75^. Therefore, adding Pr_2_​O_3_ ​to the glass system increases the power (α) from 1.026 to 1.044 as the concentration increases from 0.5 to 5.0 mol% with two different slopes indicating the nonlinearity, as presented in Table 7; Figs. 14 and 15. This value for α is near 4/3, and the (K) – (V_m_) relationship is nonlinear, suggesting a change in bonding type. This shift indicates that adding Pr_2_​O_3_ ​increases the glass’s ionic character, thereby stiffening the glass network. The observed increase in bulk modulus despite the increase in molar volume highlights the significant influence of bonding type on the bulk modulus, in addition to molar volume. This is correlated by the substitution of LiF bonds (577 kJ/mol) with stronger Pr_2_O_3_ bonds (753 kJ/mol). The introduction of stronger Pr–O bonds counteract the changes in the 4-fold tellurium atoms’ coordination number (N_4(Te)_) with increased bridging oxygens. This leads to a higher cross-link density (n_c_​), as presented in Table 5, and further confirms the increased thermal stability, and the change in bonding type. The two different rates at which the bulk modulus increases with Pr_2_O_3_​ content in the tellurite glass network corroborate this conclusion.

Moreover, the concentration of Pr_2_O_3_ correlates with increased coordination number and bond length, as well as mechanical and physical behavior of the glass network. The Te-O bond length decreased from 2.154 Å (S1) to 2.171 Å (S6), while the coordination number increased from 2.27 to 2.20 (average Te-O coordination = 2.2), as per the RDF analysis (Table 4). The Pr-O distance (∼ 2.47–2.53 Å) and coordination number (~ 5.4) indicate that Pr^3+^ ions cross-link by generating Pr-O-Te bridges, strengthening the glass network. The N_4_ values obtained from FTIR and XRD deconvolution are found to rise, demonstrating the conversion of TeO_3_ trigonal pyramidal units into TeO_4_ trigonal bipyramidal structures and increasing average network connectedness. These small structural changes are instantly apparent in macroscopic measurements. Shorter Te-O and stronger Pr-O bonds reduce molar volume, while increasing glass density, higher cross-link density and decreased mean atomic ring diameter that led to increased elastic moduli. The consistent rise in T_g_ and ΔT might be due to the structural mobility being controlled by the Te-O and Pr-O bond strengthening.

These studies all point to a clear structure-property relationship: Shorter bond lengths and a higher coordination number (N_4_) result in a more compact network with increased density, stiffness, and thermal stability.

### ANOVA hypothesis testing

The Z-distribution ANOVA hypothesis testing^76^ is applied to analyze how much the relevance of all values of each parameter to its population mean value. To determine their collective relevance, a one-way, right-tailed Z-distribution ANOVA hypothesis test is used for each parameter. The hypothesis test follows four main steps. First, the null hypothesis (H0​) and the alternative hypothesis (H1​) are stated. The null hypothesis is defined as H0: µ ≤$\:\stackrel{-}{x}$ (i.e. the values of the parameter are not different from their population mean value), where µ is the mean value of the parameter for all glass compositions under testing and $\:\stackrel{-}{x}$ is the mean value of the parameter for each separate glass composition. The alternative hypothesis is H1: µ >$\:\stackrel{-}{x}$ (i.e. the values of the parameter are different from their population mean value). The second step involves setting the significance level at 0.05, which corresponds to a critical Z-value (Z_crit_​) of −1.65. Such critical Z value corresponding to α = 0.05, has been calculated using the standard Z-distribution Table. Third, a decision rule is formulated to calculate the Z-calculated value (Z_cal_​) using the formula Z_cal_ = $\:(\stackrel{-}{x}-{\upmu\:})/(\text{S}\text{T}\text{D}\text{E}\text{V}/\sqrt{\text{n}}$, where $\:\text{S}\text{T}\text{D}\text{E}\text{V}$ is the standard deviation. Finally, the decision is made by comparing Z_cal​_ and Z_crit_​. If Z_cal_​ is less than Z_crit_​, the null hypothesis (H0) is accepted and the alternative hypothesis (H1) is rejected. Conversely, if Z_cal_​ is greater than Z_crit_​, the null hypothesis (H0) is rejected, and the alternative hypothesis (H1) is accepted. A rejection of the null hypothesis indicates that at least one of the means is different. Tables 1, 3 and 6, and 7 present the results of the hypothesis testing for all studied characteristics. For the characteristic parameters with increasing trends such as; ρ, V_m_, OPD, V_F_, G_t_, L, G, K, E, H, T_s_, n_c_, (4G/K), and α. the calculated Z_cal_ values are less than the critical value (Z_crit_​ = −1.65) in the Pr_2_O_3_​ concentration range of 0.5 to 3.0 mol%. This results in the acceptance of the null hypothesis (H0​) and the rejection of the alternative hypothesis (H1​). However, as the Pr_2_O_3_​​ concentration increases from 3.0 to 5.0 mol%, the calculated Z_cal_​ values are greater than Z_crit_​, leading to the acceptance of H1​ and the rejection of H0​. This shift confirms a structural change in the glass network at Pr_2_O_3_​ concentrations above 3.0 mol%, which is attributed to the replacement of LiF with Pr_2_O_3_​.

To analyze characteristic parameters with decreasing trends, such as U_l_, U_s_, U_m_, ℓ, n_b_, V_O_, and FPD, a one-way, left-tailed Z-distribution ANOVA hypothesis test is used. In this case, the null hypothesis is H0: µ ≥$\:\stackrel{-}{x}$, while the alternative hypothesis is H1: µ <$\:\stackrel{-}{x}$. A significance level of 0.05 is selected, with a critical Z-value (Z_crit_​) of + 1.65. The decision rule is as follows: if the calculated Z-value (Z_cal_​) is greater than Z_crit_​, H0​ is accepted and H1​ is rejected. Conversely, if Z_cal_​ is less than Z_crit_​, H1​ is accepted and H0​ is rejected. For the decreasing-trend parameters, the calculated Z_cal​_ values are found to be greater than Z_crit_​ (+ 1.65) within the Pr_2_O_3_​ concentration range of 0.5 to 3.0 mol%, confirming the acceptance of H0​ and the rejection of H1​. However, with a further increase in Pr_2_​O_3_​ concentration from 3.0 to 5.0 mol%, the calculated Z_cal_​ values are less than Z_crit_​, leading to the acceptance of H1​ and the rejection of H0​. This again confirms a structural change in the glass network at Pr_2_​O_3_​ concentrations above 3.0 mol%, which is attributed to the replacement of LiF with Pr_2_O_3_​.

Also, a one-way analysis of variance (ANOVA) with F test^76^ was performed at a 95% confidence level (α = 0.05) to determine the influence of composition on the physical and structural properties of the 60TeO_2_−12.5Nb_2_O_5_−12.5ZnO-(15-*x*)LiF-*x*Pr_2_O_3_ glass. Six glass compositions (*x* = 0.5–5.0 mol% Pr_2_O_3_) were analyzed for density, molar volume, N_4_, n_c_, elastic moduli, and T_g_. Table 8 presents ANOVA findings, indicating that all parameters have significantly larger F_(calculated)_ statistic values than F_(critical)_. This indicates that compositional changes result in statistically significant changes to structural and physical attributes. The ANOVA equation used was:

**Table 8:** Results of the one-way ANOVA F test for the parameters of the glass system under study.

Parameters	F_(calculated)_	F_(critical, α = 0.05)_	*p* value	Significance
Density (ρ)	41.17	5.05	< 0.001	Highly significant
Molar Volume (Vₘ)	12.80			
N₄	163.30			
Cross-link density (nₛ)	51.16			
Tg	34.56			
Tx	109.30			
ΔT	48.00			
T_rg_	82.40			
U_l_	50.50			
Us	37.50			
E	67.25			
G	30.00			
K	66.8			
Mean Ultrasonic Velocity (Uₘ)	21.60			
Micro-hardness (H)	43.50			
Number of bonds per unit volume (n_b_)	209.00			

4 $$\:{F}_{\left(Calculated\right)}=\frac{{SS}_{B}}{{SS}_{W}}$$

where SS_B_ and SS_W_ are the sums of squares between and within groups, respectively; k is the number of glass groups (6); and N represents the total number of measurements. The null hypothesis (H0) states that there are no differences between group means, whereas the alternative hypothesis (H1) suggests that at least one group mean is different. H0 was rejected because all examined values had a p-value of less than 0.05. All parameters show strong compositional sensitivity, especially at 3 mol% Pr_2_O_3_. Both structural (N_4_, n_c_) and physical (ρ, V_m_, T_g_) parameters show large monotonic variations. The F test analysis correlates with the Z test results suggesting a structural change in the tellurite network, including a shift from TeO_3_ to TeO_4_ coordination and improved Pr-O-Te linkages.

### Theoretically analyzed shielding properties

**Fig. 16:**
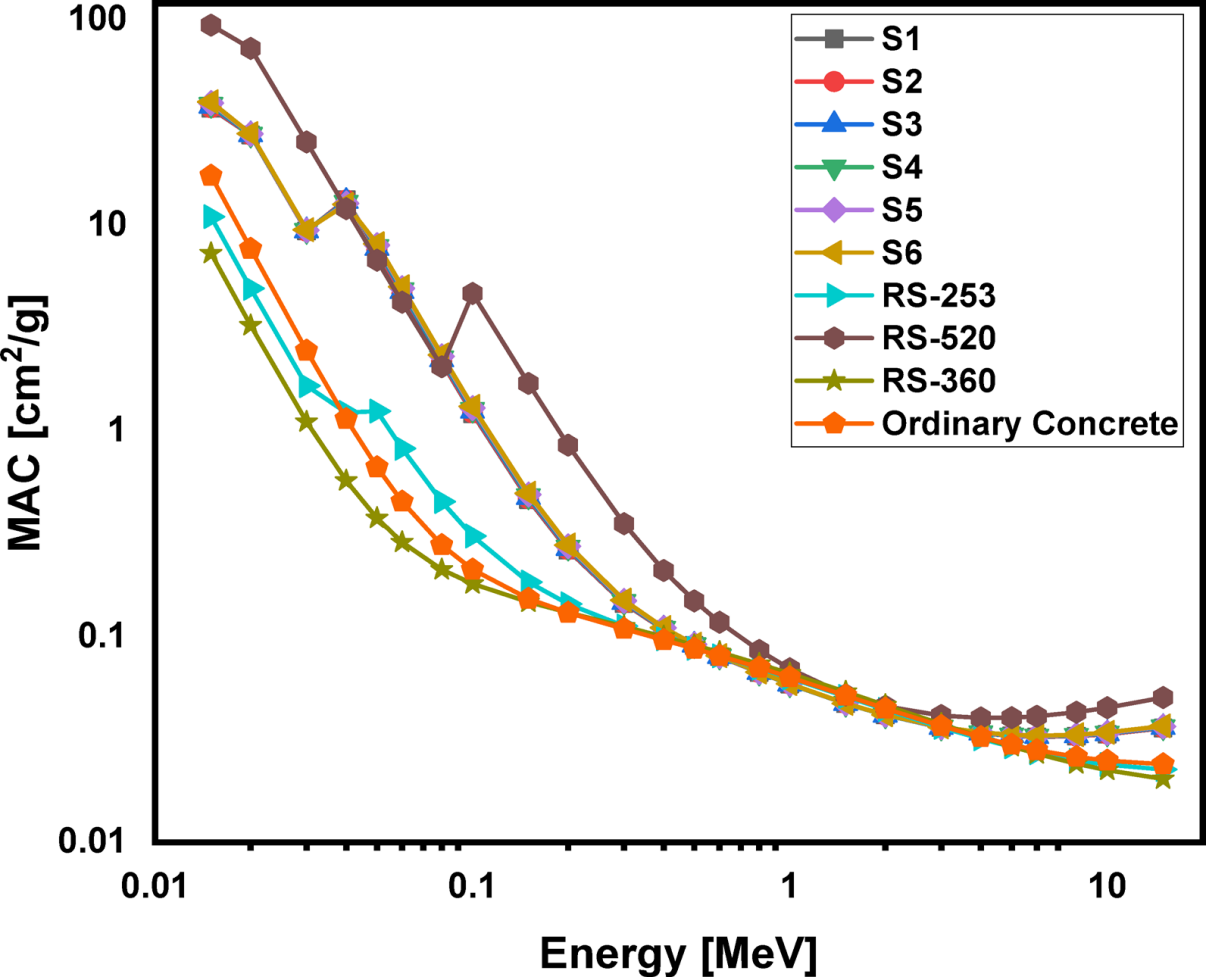
Behavior of mass attenuation coefficient (MAC) with energy for 60TeO_2_–12.5Nb_2_O_5_–12.5ZnO–(15 – *x*)LiF–*x*Pr_2_O_3_ glass system.

**Fig. 17:**
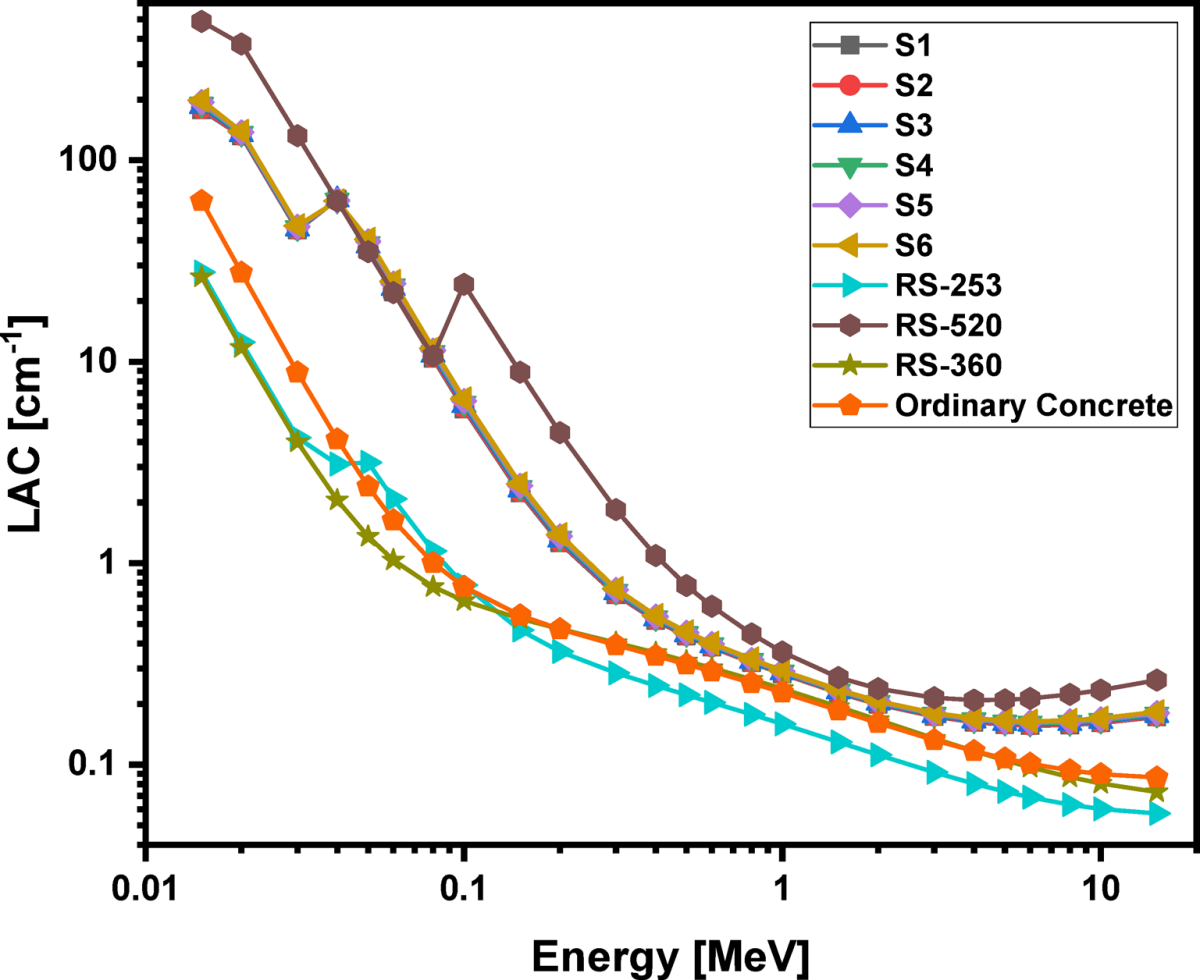
Behavior of linear attenuation coefficient (LAC) with energy for 60TeO_2_–12.5Nb_2_O_5_–12.5ZnO–(15 – *x*)LiF–*x*Pr_2_O_3_ glass system.

**Fig. 18:**
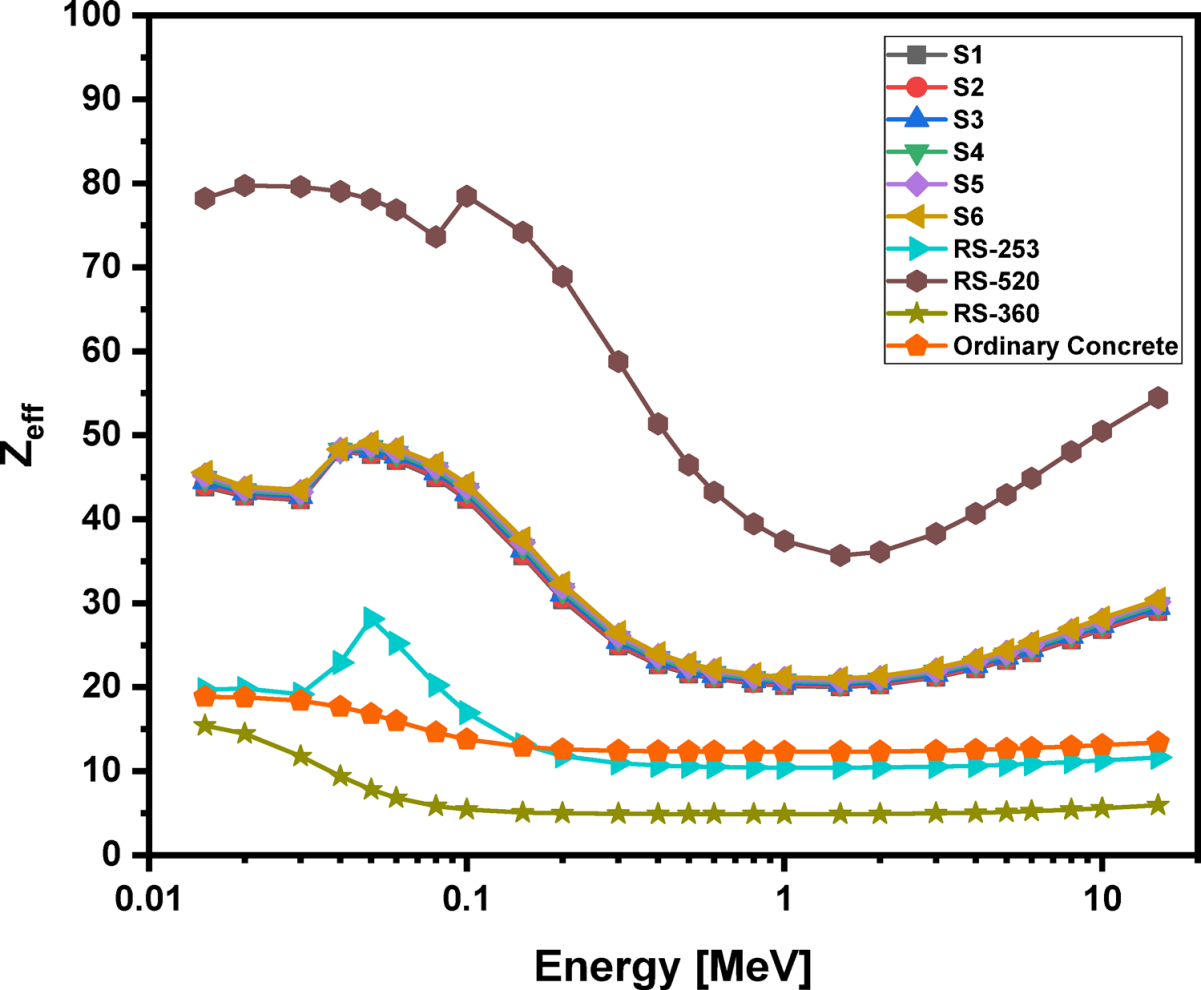
Behavior of effective atomic number (Z_eff_) with energy for 60TeO_2_–12.5Nb_2_O_5_–12.5ZnO–(15 – *x*)LiF–*x*Pr_2_O_3_ glass system.

**Fig. 19:**
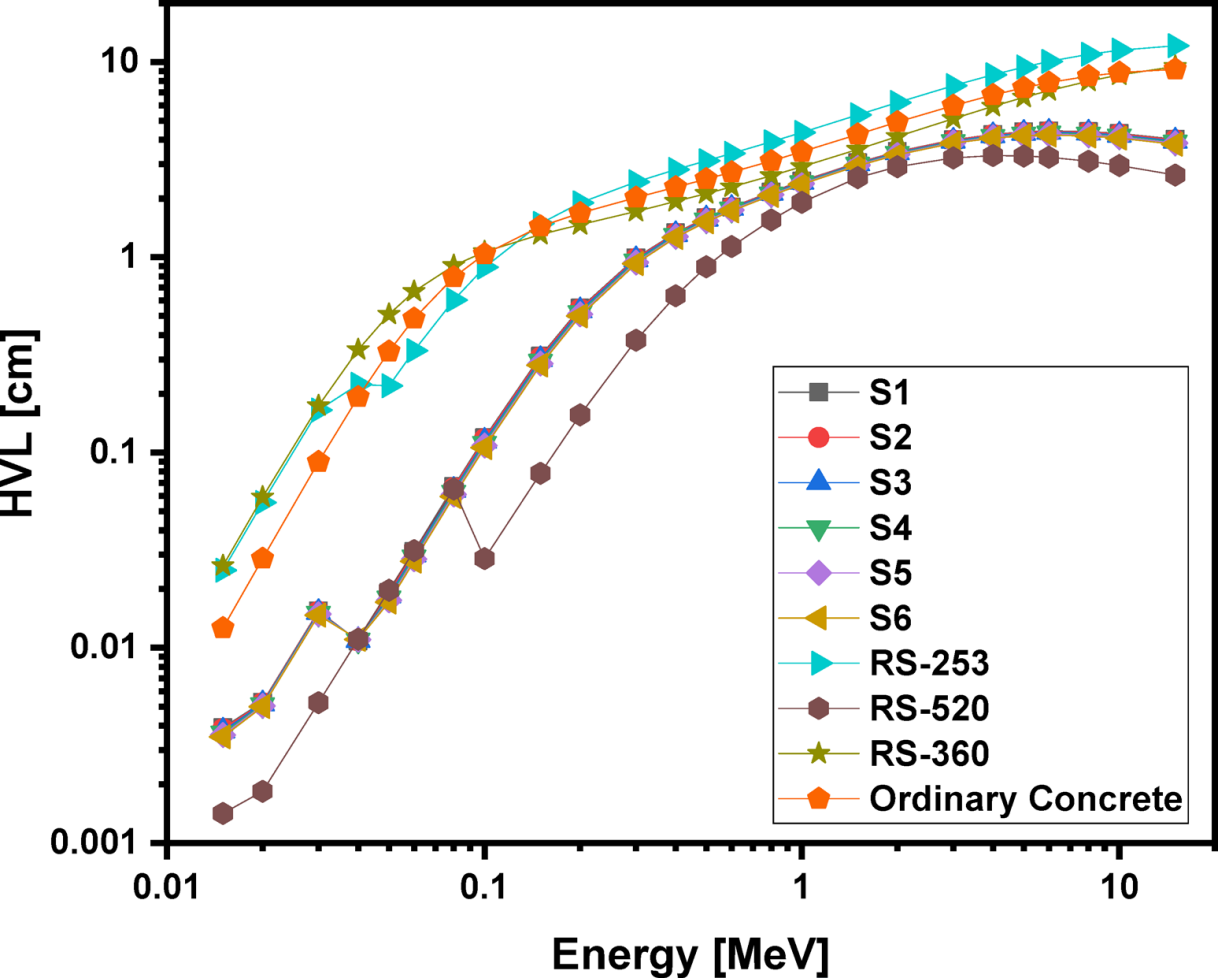
Behavior of half value layer (HVL) with energy for 60TeO_2_–12.5Nb_2_O_5_–12.5ZnO–(15 – *x*)LiF–*x*Pr_2_O_3_ glass system.

 Using the phy-x computer program, the radiation protection parameters for the glass samples are accurately calculated. Figures 16, 17, 18 and 19 illustrate the spectra for the mass attenuation coefficient (MAC), linear attenuation coefficient (LAC), effective atomic number (Z_eff_​), and half-value layer (HVL), respectively. The (MAC) results demonstrate that the Pr^3+^ ions are identified by the K-absorption edge at approximately 0.04 MeV. The value of the MAC is evaluated to be lowest (36.960 cm^2^/g) for the S1 glass at 0.015 MeV photon energy and highest (39.866 cm^2^/g) for the S6 glass at 0.015 MeV photon energy. The gradual increase in Pr^3+^ ions with higher atomic number replaces Li^2+^ ions within the glassy network, increasing the physical characteristics, which further lead to the rise in MAC values. These changes are further impacted by the enhancement of bridging oxygen (BO) sites during the process of glass formation. The same behaviors were observed for (LAC), and (Z_eff_), and their results show that the studied glass system has higher values of MAC, LAC, and Z_eff_​ compared to ordinary concrete and commercial shielding glasses like RS-253 and RS-360. However, its values of MAC, LAC, and Z_eff_​ are slightly lower than those of the commercial glass RS-520, which contains 71 mol% of the heavy metal oxide PbO. Furthermore, as shown in Fig. 19, our glass has a lower HVL for blocking gamma rays than ordinary concrete, RS-253, and RS-360, but a slightly higher HVL than RS-520. These findings indicate that the studied echo-friendly glass system possesses excellent gamma ray shielding properties, aligning with previously published studies^11,25,26,77–87^.

A comparison of the current Pr_2_O_3_-modified tellurite glasses with other documented TeO_2_-based systems including boro-, heavy-metal-, and rare-earth-modified compositions is shown in Table 9. The investigated series has high thermal characteristics (Tg ≈ 622–637 K; ΔT = 134.6–159.2 K) and densities of 4.80–4.97 g.cm^− 3^, which are on the same level with or greater than the majority of tellurites that include lead and bismuth. The improved mechanical rigidity and glass stability are explained by the gradual rise in the proportion of tetrahedral TeO_4_ units (N_4_ = 0.41–0.53), which validates increased network polymerization and cross-link density. The Pr_2_O_3_-doped glasses preserve a lead-free composition while achieving comparable effective atomic numbers (Z_eff_ ≈ 52–56) and mass attenuation coefficients (0.086–0.094 cm².g⁻¹ at 0.662 MeV) to heavy-metal systems like PbO- or Bi_2_O_3_-rich tellurites. These glasses are among the most effective environmentally friendly gamma-shielding materials known to date due to its half-value layer (1.80–1.72 cm). Further evidence that Pr^3+^ functions as a multifunctional structural stabilizer by simultaneously strengthening the Te–O–Te network and improving photon attenuation capabilities comes from comparison with HfO_2_, WO_3_, and other rare-earth-modified systems. The current Pr_2_O_3_ tellurite glasses combine high structural compactness, superior thermal endurance, and effective radiation-shielding efficiency, making them promising candidates for advanced photonic and protective glass applications, according to the correlations summarized in Table 9.

**Table 9:** Comparison of Pr_2_O_3_–tellurite glasses’ structural, thermal, and gamma-shielding characteristics with those of systems that have been reported.

Glass system	Density (g cm⁻³)	Tg (K)	ΔT (K)	*N*₄	Zeff	MAC (cm² g⁻¹ @ 0.662 MeV)	HVL (cm @ 0.662 MeV)	Reference
60TeO₂–12.5Nb₂O₅–12.5ZnO–(15–x)LiF–xPr₂O₃ (Present study)	4.80–4.97	622–637	134–159	0.41–0.53	52–56	0.086–0.094	1.80–1.72	This work
TeO₂–B₂O₃–WO₃–HfO₂	4.68–4.83	690–715	60–65	0.38–0.42	49–51	0.091–0.098	1.40–1.45	^67^
B₂O₃–TeO₂–Bi₂O₃–CdO–Tm₂O₃	5.00–5.15	700–730	60–70	0.39–0.45	54–57	0.093–0.101	1.32–1.37	^88^
B₂O₃–TeO₂–BaO–PbO–V₂O₅	5.10–5.25	695–725	55–65	0.40–0.46	55–58	0.095–0.102	1.30–1.35	^89^
TeO₂–Bi₂O₃–ZnO–Na₂O	4.95–5.10	675–700	50–55	0.40–0.45	53–55	0.090–0.097	1.35–1.40	^90^

## Conclusion

The thermal, mechanical, gamma shielding, and structural properties of the 60TeO_2_–12.5Nb_2_O_5_–12.5ZnO–(15–*x*)LiF–*x*Pr₂O₃ (0.5 ≤ *x* ≤ 5.0 mol%) glass system have been investigated in detail. The following are the primary points of this work:

1. When Pr_2_O_3_ is added, RDF and FTIR measurements reveal that the coordination of the structural units shifts from TeO_3_ (trigonal pyramidal) to TeO_4_ (trigonal bipyramidal). As a result of this modification, the tellurite network polymerizes more effectively by adding more bridging oxygens (BOs). The increasing cross-link density (n_c_ = 2.30 to 2.41) and N_4_ values (from 0.41 to 0.53) indicate that the structure is becoming more compact and rigid.

2 According to the RDF interatomic lengths (Pr-O ≈ 2.46 Å), Pr^3+^ ions form strong Pr-O-Te bonds instead of weaker Li-F interactions. With the use of the ring deformation model, the average atomic ring diameter (ℓ = 5.39 to 5.18 nm) decreased. In other words, there was less vacant space and the glass matrix was packed more densely.

3 The results of differential thermal analysis (DTA) showed that the amount of Pr_2_O_3_ causes an incremental increase in the glass transition temperature (T_g_ = 622.8 to 632 K), the start of crystallization (T_x_ = 757 to 791 K), and thermal stability (ΔT = 134.6 to 159.2 K). The lower glass transition ratio (T_rg_ = 0.519 to 0.564) indicates that the material is less likely to crystallize. E = 59.4 to 59.8 GPa and K = 36.4 to 36.6 GPa, the calculated elastic moduli, support the notion that the material is stiffer and has more network connections.

4 Variations in density, molar volume, N_4_, n_c_, and T_g_ across compositions were confirmed to be statistically significant to the one-way ANOVA test. In the system under study, this demonstrates that the primary factor influencing the structure-property connection is the concentration of Pr_2_O_3_.

5 The half-value layers (HVL ≈ 1.80–1.72 cm at 0.662 MeV) are lowered by the addition of Pr_2_O_3_, which improves the mass attenuation coefficients (MAC), effective atomic number (Z_eff_ = 52–56), and linear attenuation coefficients (LAC). These results suggest that the tested glasses are environmentally benign, lead-free, and on par with commercial shielding materials such as RS-253 and RS-360.

6 It can be shown from the logarithmic relationship between molar volume (V_m_) and experimental bulk modulus (K) that increasing the concentration of Pr_2_O_3_ simultaneously makes the material more compact and rigid. Structure, property, and shielding are clearly related, as shown by the combined analysis of thermal, gamma, and structural attenuation characteristics. This demonstrates that in tellurite glasses, Pr_2_O_3_ functions as a multipurpose modifier.

The Pr_2_O_3_ tellurite glass system is very dense, mechanically rigid, thermally stable, and effective in shielding radiation. For sophisticated photonic, optoelectronic, and radiation-protective applications, these qualities make it a solid option. It works well to replace conventional heavy-metal oxide glasses without the use of lead.

## Data Availability

The datasets generated and/or analyzed during the current study are not publicly available because all data are presented in the article and therefore, there is no need to include raw data, but they are available from the corresponding author upon reasonable request.
